# Systems modeling of oncogenic G-protein and GPCR signaling reveals unexpected differences in downstream pathway activation

**DOI:** 10.1038/s41540-024-00400-1

**Published:** 2024-07-16

**Authors:** Michael Trogdon, Kodye Abbott, Nadia Arang, Kathryn Lande, Navneet Kaur, Melinda Tong, Mathieu Bakhoum, J. Silvio Gutkind, Edward C. Stites

**Affiliations:** 1 Integrative Biology Laboratory, Salk Institute for Biological Studies, La Jolla, CA 92037 USA; 2 Department of Laboratory Medicine, Yale School of Medicine, New Haven, CT 06520 USA; 3 Moores Cancer Center, University of California, San Diego, La Jolla, CA 92093 USA; 4 Biomedical Sciences Graduate Program, University of California, San Diego, La Jolla, CA 92093 USA; 5 Razavi Newman Integrative Genomics and Bioinformatics Core, Salk Institute for Biological Studies, La Jolla, CA 92037 USA; 6 Department of Ophthalmology and Visual Science, Yale School of Medicine, New Haven, CT 06520 USA; 7 Yale Cancer Center, Yale School of Medicine, New Haven, CT 06520 USA; 8 Department of Pharmacology, University of California, San Diego, La Jolla, CA 92093 USA; 9 Present Address: Pfizer, La Jolla, CA 92037 USA

**Keywords:** Biochemical networks, Cancer, Computational biology and bioinformatics

## Abstract

Mathematical models of biochemical reaction networks are an important and emerging tool for the study of cell signaling networks involved in disease processes. One promising potential application of such mathematical models is the study of how disease-causing mutations promote the signaling phenotype that contributes to the disease. It is commonly assumed that one must have a thorough characterization of the network readily available for mathematical modeling to be useful, but we hypothesized that mathematical modeling could be useful when there is incomplete knowledge and that it could be a tool for discovery that opens new areas for further exploration. In the present study, we first develop a mechanistic mathematical model of a G-protein coupled receptor signaling network that is mutated in almost all cases of uveal melanoma and use model-driven explorations to uncover and explore multiple new areas for investigating this disease. Modeling the two major, mutually-exclusive, oncogenic mutations (Gα_q/11_ and CysLT_2_R) revealed the potential for previously unknown qualitative differences between seemingly interchangeable disease-promoting mutations, and our experiments confirmed oncogenic CysLT_2_R was impaired at activating the FAK/YAP/TAZ pathway relative to Gα_q/11_. This led us to hypothesize that *CYSLTR2* mutations in UM must co-occur with other mutations to activate FAK/YAP/TAZ signaling, and our bioinformatic analysis uncovers a role for co-occurring mutations involving the plexin/semaphorin pathway, which has been shown capable of activating this pathway. Overall, this work highlights the power of mechanism-based computational systems biology as a discovery tool that can leverage available information to open new research areas.

## Introduction

Data intensive computational methods have contributed to significant advances in a variety of scientific disciplines. Cancer biology in particular has seen many advances that follow from the application of computational methods to emerging forms of acquired data^1–6^. However, not all cancers have abundant data. Rare cancers, and rare subtypes of cancer, do not generally have large data sets of the scale required for many computational methods. The number of patient samples, tumor derived cell lines, and mouse models that can be used to generate new, large data sets are commonly also limited for rare cancers. However, years of broad research efforts for individual rare cancers have resulted in several areas where modest amounts of data are available. For example, cell biological, biochemical, genomic (DNA), and transcriptomic (RNA) characterizations are frequently available for rare cancers. We hypothesized that biochemical-mechanism based models of the essential molecular network(s) in a rare cancer may provide a useful approach for re-analyzing the available data to generate new inferences and insights.

Previously, we have used mathematical modeling to investigate signaling by pathogenic RAS pathway mutations^7–13^. In these previous studies, we simulated the core biochemical processes that regulate RAS signaling. The model parameterized for wild-type proteins describes the dynamic equilibrium of signaling in a resting cell. Mutant versions of RAS proteins are characterized by changes in these biochemical rate constants and elevated levels of steady-state signaling^14–16^. By substituting biochemical parameters that involve the reactions of a given protein with the biochemical kinetic parameters (e.g., k_f_, k_r_, k_cat_, K_m_, etc.) the model predicts how signaling outcomes differ when the mutant is present^17–19^. This approach has been useful for uncovering new aspects of RAS mutant biology^7,8^, for solving long-standing questions about RAS mutant biology^10,11,20^, and for generating new ideas about targeting the pathway^7,9,12,13^. This general approach has been reproducibly used by different groups to study oncogenic RAS signaling^7,14,16,21–23^, attesting to the reproducible nature of the general computational approach even if the specific details of the model differ from group to group (just as different experimental groups may utilize slightly different reagents or methods for otherwise conceptually similar experiments). Moreover, the approach of comparing physiological signaling (from wild-type proteins) with pathological signaling (by mutant proteins) has been broadly used for diseases other than cancer and/or for proteins other than RAS^24–32^, further highlighting the potential power of the method to be generalized and applied more extensively. Previous work has also highlighted that mathematical models of a modest scope (approximately five to twenty-five molecular species) can be generated and used to formulate novel hypotheses about pathways important to disease on a time-scale comparable to experimental screens (~6 months)^33^. Altogether, the previous studies suggest that mathematical models of disease-causing mutations in signaling networks could potentially be useful as a discovery tool that can open new areas of research for subsequent validation of model-based inferences and predictions.

To do this, we combine mechanism-based computational modeling with bioinformatic analysis and experimental biology to investigate uveal melanoma (UM), a form of cancer that has an incidence of approximately five per million individuals in the United States^34^. We focused on UM as a test case because there are much less data for UM than for more common cancers, because of the importance of G-protein coupled receptor signaling in UM, and because oncogenic G-proteins have proven well-suited for mechanistic modeling. We first developed a biochemical mechanism-based mathematical model of the biomolecular signaling pathway that is mutated and plays a causal role in nearly all cases of UM. As part of our model validation, we demonstrate our model can explain confusing aspects of recently developed direct pharmacological inhibitors against the major driver of this cancer^35–38^. Next, we modeled and compared two major classes of UM. Our mechanistic model revealed gaps in available knowledge that are of fundamental importance to understanding the molecular basis of this disease. We addressed these existing gaps in knowledge with new experiments and we updated our model to the new information. These insights led us to hypothesize that there may be another pathway that augments the known pathways driving UM. Evaluation of the available, limited, DNA sequencing data with our focused hypothesis in mind led us to identify statistically enriched mutations in the plexin/semaphorin signaling pathway. Overall, our study demonstrates that this integrated approach, which we call MAGPIE for Model-Assisted Generation of Predictions and Integration of Experimental Data, can catalyze scientific progress through its ability to leverage multiple “modest data” resources.

## Results

### Development of a mechanistic mathematical model of oncogenic GPCR-pathway signaling in UM

Uveal melanoma (UM) is a rare but deadly cancer of the eye^34^. Although both UM and cutaneous melanoma arise from melanocytes, the two malignancies are biologically and genetically distinct^39^. Despite the great progress made in recent years in the development of both targeted therapies and immunotherapies for cutaneous melanoma^40^, there are no FDA approved targeted therapies for UM^39^ and only one FDA approved immunotherapy^41^. The identification of new pathways and targets for the treatment of UM thus remains a priority.

**Fig. 1:**
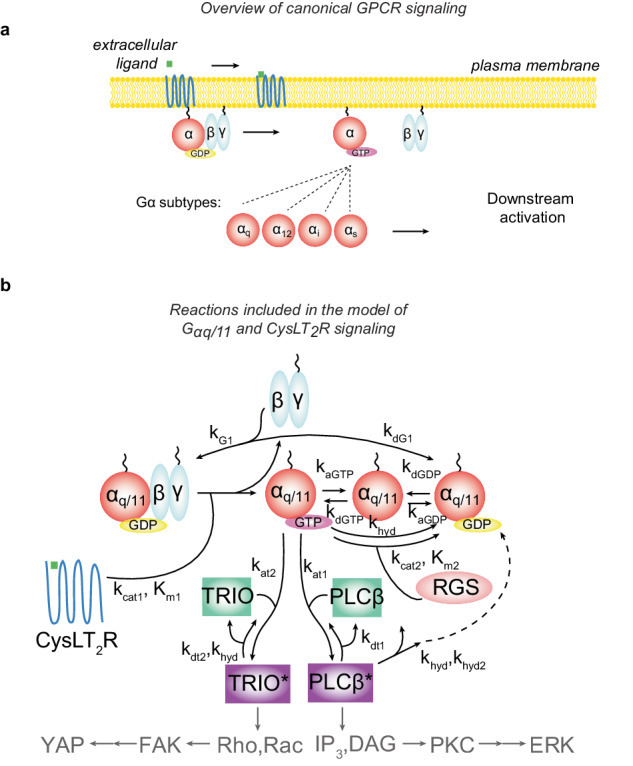
A mass-action kinetics-based dynamic equilibrium mathematical model of Gα_q/11_ and CysLT_2_R signaling in UM. **a** Schematic of canonical G protein-coupled receptor (GPCR) signaling. **b** Schematic of the mechanistic mathematical model of Gα_q/11_ and CysLT_2_R signaling in uveal melanoma (UM) with chemical kinetic parameter constants annotated. Within the G protein activation/inactivation cycle, CysLT_2_R is the guanine nucleotide exchange factor (GEF) that activates the small G-proteins Gα_q_ and Gα_11_ by promoting the replacement of bound guanosine diphosphate (GDP) for guanosine triphosphate (GTP), regulator of G protein signaling (RGS) is the GTPase-activating protein (GAP) that inactivates the small G-proteins Gα_q_ and Gα_11_ by promoting the hydrolysis of bound GTP to GDP, and both TRIO and PLCβ are effectors that bind GTP-bound Gα_q_ and Gα_11_. Within (**a**, **b**), the schematized G-protein alpha subunits (red circles) can represent either (or both) wild-type and mutant forms.

UM is highly dependent upon G-protein coupled receptor (GPCR) signaling (Fig. 1a). GPCRs are one of the largest and most diverse families of membrane receptors in the genome^42^. GPCRs bind a wide variety of extracellular ligands which result in conformational changes of the receptor. Activated GPCRs catalyze the exchange of GDP for GTP in the alpha subunits (Gα) of heterotrimeric G-proteins downstream. Once bound to GTP, the Gα subunits effectively disassociate from the beta-gamma subunits (Gβγ) to bind and activate effectors downstream. Evaluation of the somatically acquired mutations in UM have highlighted a critical role for one GPCR pathway. Mutually exclusive mutations in the *CYSLTR2*/*GNAQ*/*GNA11*/*PLCB4* signaling pathway occur in >95% of UM cases^39^. Mutations to the Gα encoding genes *GNAQ* and *GNA11* (which will be referred to together as *GNAQ/11*) account for the overwhelming majority (~90%) of these mutations in UM^43^.

The most frequent *GNAQ/11* mutations occur in a hotspot at codon 209. Two of these mutations, *GNAQ/11* Q209L and Q209P, have been shown to encode mutant Gα subunits that differ in key biochemical properties, including binding to effector proteins and to regulator of G protein signaling (RGS) proteins. RGS proteins are the GTPase-activating proteins (GAPs) that catalyze the conversion of GTP-bound Gα_q/11_ to GDP-bound Gα_q/11_^44^. Although there are significant differences in several of the biochemical properties of WT and Q209L/P mutant Gα_q_ subunits, the importance of these various differences for tumorigenesis in vivo remains unclear. Mathematical modeling of GPCR signaling pathways has a substantial history of aiding in understanding the dynamics of GPCRs in response to ligands and pharmacological targeting^45–47^. We here developed a mechanistic mathematical model of the Gα_q/11_ pathway that begins at the level of the GPCR CysLT_2_R (the protein encoded by the *CYSLTR2* gene), that includes the G-protein activation cycle, and that includes the binding to its two primary effector proteins, PLCβ and TRIO^48,49^ (Fig. 1b). The activation of CysLT_2_R by ligand and the transmission of signals downstream from PLCβ and TRIO are not included explicitly in the model (see below for more details).

Some key features of the proposed model are as follows: first, the reactions are modeled mechanistically using ordinary differential equations (ODEs). This mathematical formalism is based on mass action and classical enzyme kinetics. Direct measurements of kinetic rate constants for the various reactions were taken from the literature wherever possible. Second, a key component of the model is that when considering a particular oncogenic mutation (i.e., the Q209L mutation in Gα_q_) this mutant protein is considered explicitly as a distinct species in the model with its own biochemical properties. For example, to model the case of a heterozygous *GNAQ* Q209L mutation, of the total pool of Gα subunits in the model, 75% will be WT and 25% will be mutant (this reflects an assumption of the model that the Gα_q_ and Gα_11_ subunits are biochemically identical and expressed in similar amounts in vivo, thus Gα_q_ and Gα_11_ each account for 50% of the total Gα pool in the model). The main biochemical differences between WT and mutant Gα_q_ subunits reported in the literature are the rates of basal and GAP-stimulated GTP hydrolysis and the affinities for effectors and GAPs (the supplement includes a full discussion of the literature concerning biochemical characterization of various mutants). For model parameterization, we initiate our study by identifying a set of approximate values for each parameter based upon the relevant literature (see the supplement for details). Additional mathematical details, references for the biochemical parameters of the model, and a Python notebook that includes all of the code needed to run and reproduce the model are included as supplementary information and/or on github.

While oncogenic activation ultimately depends on downstream targets, such as Yes-associated protein (YAP) for TRIO and the Extracellular Signal-Regulated Kinase (ERK) Mitogen Activated Protein Kinase (MAPK) pathway via protein kinase C (PKC) for PLCβ, the model remains agnostic to these outcomes by focusing specifically on the part of the pathway mutated in UM. Activation of both downstream pathways, ERK and YAP, by oncogenic driver mutations in the *CYSLTR2/GNAQ/GNA11/ PLCB4* pathway has been identified as critical for the clinical pathogenesis of UM, and combinations of inhibitors targeting these pathways are currently in early phase clinical trials^50^. One key aspect of PLCβ that we include in our model is that, in addition to acting as a Gα_q/11_ effector protein that can transmit signals downstream, PLCβ can also act as a GAP for Gα_q/11_^51^. Lastly, while the time dynamics of signaling pathways are critical in certain circumstances, here we focus on the steady-state activation of effectors because in cancer it is the sustained levels of growth signals that seem to be most relevant. Thus, we focus on the dynamic equilibrium that occurs at steady state. As we do not explicitly model signaling downstream of PLCβ and TRIO, we implicitly assume that there is a monotonic relationship between each of these effectors and their downstream pathways (i.e., increased TRIO activation results in increased Rho/Rac/FAK/YAP signaling, and increased PLCβ activation results in increased IP_3_/DAG/PKC/ERK signaling). Similarly, the model assumes a small basal level of receptor in the active conformation^52^ that implicitly takes the steady-state levels of β-arrestin desensitization into account. While the activation of a GPCR by its ligand can involve a complex set of intramolecular reconfigurations and is an important aspect of canonical G-protein signaling in response to time-varying ligand concentrations, here we focus on the steady-state activation levels relevant in oncogenic signaling. Thus, the main functional outputs for the model are the steady-state levels of activated TRIO and PLCβ (by either WT or mutant Gα subunits).

In summary, the model presented here attempts to capture the following key underlying biochemical steps: basal active CysLT_2_R catalyzes the exchange of GDP for GTP in the alpha subunits (Gα_q/11_) of heterotrimeric G-proteins downstream. Once bound to GTP, the Gα_q/11_ subunits effectively disassociate from the beta-gamma subunits (Gβγ). Once disassociated, the GTP-bound Gα_q/11_ can either directly bind and activate one the downstream effectors PLCβ or TRIO, disassociate from GTP to become nucleotide-free Gα_q/11_ or catalyze the hydrolysis of GTP to GDP through either intrinsic GTPase activity or through the catalyzed action of RGS. The cycle is then completed when GDP-bound Gα_q/11_ again binds Gβγ to form the heterotrimeric G-protein. The mutant CysLT_2_R and Gα_q/11_ proteins are modeled explicitly as distinct species in the model that undergo the same reactions described here but with different biochemical properties compared to the WT proteins based on available data.

### The mathematical model reproduces key signaling outputs for different modeled disease genotypes

**Fig. 2:**
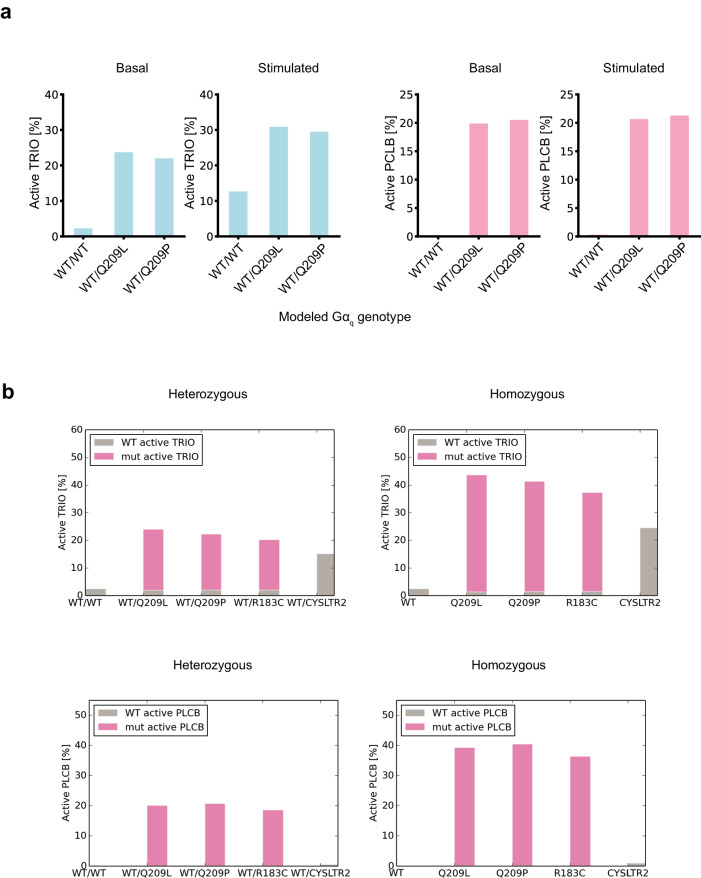
Characterization of the kinetics-based dynamic equilibrium mathematical model of Gα_q/11_ and CysLT_2_R signaling in UM. **a** Active TRIO and PLCβ levels resulting from simulation of the mathematical model for cases where all *GNAQ/11* is WT, where one GNAQ/11 allele is the oncogenic Q209L, and where one GNAQ/11 allele is the oncogenic Q209P allele for modeled basal conditions (no CysLT_2_R ligand-mediated activation) and for modeled stimulated conditions (CysLT_2_R ligand-mediated activation). **b** Active TRIO and PLCβ levels resulting from simulation of the model for modeled heterozygous (one copy) and homozygous (two copies) of *GNAQ/11* Q209L, *GNAQ/11* Q209P, *GNAQ/11* R183C, and *CYSLTR2* L129Q compared to the modeled all *GNAQ/11* and *CYSLTR2* WT genotype.

As a first evaluation of the mathematical model described above, we simulated the common Gα_q/11_ mutants: Q209L and Q209P. The Q209L/P mutants were considered heterozygous (i.e., 75% of Gα subunits are modeled as WT and 25% mutant, as described above). These assumptions are based on the TCGA data from cBioPortal which show that *GNAQ* Q209L/P mutations are typically heterozygous^53,54^. The model naturally and emergently results in significantly higher steady-state levels of complexes between activated (GTP-bound) Gα and its effectors TRIO and PLCβ (hereafter, “activated TRIO” and “activated PLCβ”) for each of the mutant Gα_q_ cases when compared to the basal WT case (Fig. 2a). This suggests that the mechanisms considered by our mathematical model, when combined with approximate parameters for the WT and mutant proteins, are sufficient to explain the activation of these mutants.

We considered the anticipated behavior if the oncogenic mutation was homozygous instead of heterozygous. Modeling this case, where there was now twice as much of the mutant protein abundance in the model but the same total amount of wild-type plus mutant Gα_q/11_, resulted in a slight increase in total steady-state signal of activated TRIO and activated PLCβ (Fig. 2b). We extended our model to include the less strongly activating R183C Gα_q/11_ mutations. Introduction of the approximate mutant parameters also resulted in constitutive signaling of the pathway for this mutant in both heterozygous and homozygous cases (Fig. 2b).

### The mathematical model reproduces unanticipated responses to pharmacological G-protein inhibition

One of the major goals of understanding the molecular causes of tumorigenesis in UM is to develop better therapies. Most efforts in the clinic up to this point have focused on direct inhibition of downstream effectors such as MEK and PKC or on an immunotherapy approach. Both general approaches have had limited success^39^.

One key development in the field over the last several years has been the discovery and exploration of direct pharmacological inhibitors of Gα_q/11_ subunits such as YM-254890 (YM) and the naturally occurring cyclic depsipeptide FR900359 (FR)^35–38^. Specifically, FR has been shown to inhibit Gα_q/11_ signaling in UM cells and mouse xenograft models^37,38^. The current consensus is that FR acts as a guanine nucleotide disassociation inhibitor (GDI), effectively locking Gα _q/11_ in the GDP-bound, inactive state, although it is unclear if this is the only mechanism responsible for the inhibition of oncogenic signaling observed in various in vitro and in vivo models of UM^38^. While it has been recently shown that the major effects of FR/YM are in fact on-target for Gα_q/11_^55^, the efficacy of a drug that targets the inactive (GDP-bound) form of a mutant protein that is “constitutively active”, where the active form is GTP-bound, has created confusion about this mechanism of action. This confusion regarding mechanism is similar to the issues originally surrounding the direct targeting the KRAS G12C oncoprotein with inhibitors that target the GDP-bound form of the constitutively active KRAS G12C mutant^9,56–58^.

**Fig. 3:**
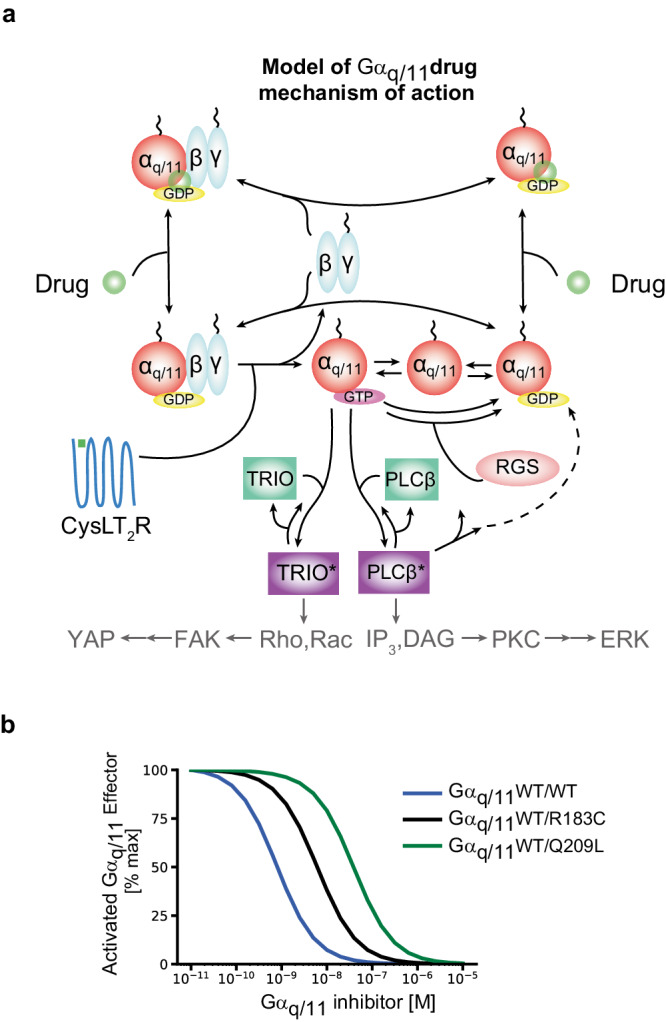
Extension of the model to Gα_q/11_ inhibition. **a** Schematic of extensions to the model presented in Fig. 1b to include a Gα_q/11_-inhibitor. The schematized G-protein alpha subunits (red circles) can represent either (or both) wild-type and mutant forms. **b** Model simulations of Gα_q/11_-inhibitor dose responses on modeled GNAQ/11 WT, heterozygous R183C, and heterozygous Q209L genotypes.

As another test of whether mathematical modeling can be useful in situations that are confusing to domain experts of the biological subject matter, we expanded the model to include drug targeting. Specifically, we modeled a FR/YM-type GDI drug that can reversibly bind and sequester GDP-bound Gα subunits (Fig. 3a). We then simulated the model at varying drug concentrations for both WT/WT and WT/mutant cases (Fig. 3b). The model suggests the proposed FR mechanism of binding to the GDP-bound form of the “constitutively active” Gα mutants is sufficient to explain its action. In other words, modeling reveals that the issues that superficially appeared confusing are not actually problematic and do not indicate a gap in mechanistic understanding. Interestingly, the model is able to replicate a reported^59^ log-shift in sensitivity between Gα_q_ mutants that are known to be partially GAP-sensitive (e.g., R183Q/C)^60^ and more common oncogenic mutants such as Gα_q_ Q209L (Fig. 3b). The model’s ability to predict relative differences in response to pharmacological inhibition, including the ability to reproduce the non-intuitive effects of a drug that binds selectively to GDP-bound forms of Gα_q/11_, help validate our mathematical model and suggest it may have future value to help contextualize and interpret drug development studies for UM.

### Mathematical model illuminates unknown differences in downstream signal activation between mutant Gα_q_ and mutant CysLT_2_R

**Fig. 4:**
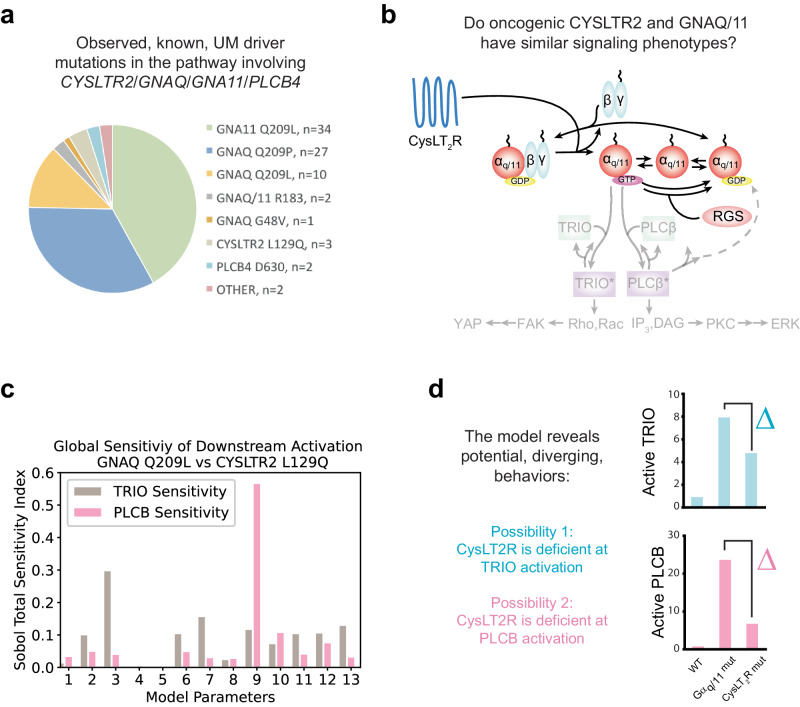
Mathematical model predicts potential for major differences in downstream activation for *GNAQ* and *CYSLTR2* mutations. **a** Pie chart of mutations in the pathway involving *CYSLTR2*/*GNAQ*/*GNA11*/*PLCB4* for 80 UM patients reported in previous studies^53,54^. **b** Schematic of oncogenic Gα_q_ and oncogenic CysLT_2_R signaling in the model. The schematized G-protein alpha subunits (red circles) can represent either (or both) wild-type and mutant forms. **c** Sobol total sensitivity index for the difference in active TRIO and PLCβ between the *GNAQ* Q209L and *CYSLTR2* L129Q mutant settings based on simulation of the mathematical model. See the supplement for details on the parameters varied and ranges. **d** Schematic highlighting two diverging behaviors suggested from the extended simulations of the model across parameter space: (1) that CysLT_2_R mutant networks are less able to activate TRIO/YAP/TAZ than Gα_q/11_ mutant networks, or (2) that CysLT_2_R mutant networks are less able to activate PLCβ/PKC/ERK than Gα_q/11_ mutant networks. Which situation occurs will depend on where the actual system is within parameter space, and could be determined by experimentally measuring parameters and/or by experimentally measuring cellular phenotypes. The bar charts on the right display representative sample cases of the two diverging behaviors; fold change in activation relative to the WT case is used as a measure of pathway activation within these bar charts.

UM cases without *GNAQ/GNA11* mutations often contain activating mutations upstream in the associated receptor CysLT_2_R^61,62^ or in the canonical downstream effector of Gα_q_, PLCβ4 (the protein encoded by PLCB4)^63^ (Fig. 4a). Most of the genomic data on UM is for *GNAQ*/*GNA11* mutant cases and most clinical data is from patients with a *GNAQ*/*GNA11* mutation. Additionally, while there are several preclinical experimental model systems to study *GNAQ/GNA11* mutant UM, from cell lines to mouse models^64^, there are no equivalent experimental model systems for *CYSLTR2* mutant UM^65^. Although the biochemical properties have been measured for the most common point mutations, Q209L and Q209P, in Gα_q/11_^44^, similar biochemical data for point mutations in CysLT_2_R (the upstream receptor that activates Gα_q_) are not available. We hypothesized that, despite the gaps in knowledge, our mechanistic model mapping between biochemical properties and cellular phenotypes may be able to clarify ranges of potential behaviors and be able to generate insights that prioritize new areas for experimental and/or bioinformatic investigation.

We updated our model to the most common CysLT_2_R mutation: L129Q (Fig. 4b). This mutation was taken to be heterozygous based on TCGA data and modeled as a higher surface concentration of activated receptor (i.e., 50% of the CysLT_2_R receptor population was modeled as WT and 50% mutant). Recent work has demonstrated that the CysLT_2_R L129Q mutant poorly recruits β-arrestin to avoid desensitization^66^. Current opinion in the field is that the mutually exclusive *GNAQ/11* and *CYLSTR2* mutations provide similar oncogenic signals, and our initial parameterization estimates based on the best available data for the CysLT_2_R L129Q mutant resulted in an elevation of active TRIO that was slightly to moderately weaker than what the model predicts for Gα_q/11_ mutations (Fig. 2b). However, we also noticed that our initial parameter estimates resulted in a near complete loss of PLCβ activation compared to the Gα_q/11_ mutations (Fig. 2b). We hypothesized that this might suggest our initial parameter estimates need to be adjusted.

To further explore the potential ranges of behaviors with the model for alternative parameter sets, we evaluated the potential similarity of model-predicted behaviors for CysLT_2_R and Gα_q/11_ mutant conditions over a wide range of concentrations of the proteins considered in the model and over a wide range of kinetic rate constants for the considered reactions. For each parameter set, we simulated our mathematical model of the Gα_q/11_ Q209L and CysLT_2_R L129Q mutations. We then performed a global sensitivity analysis to compare the downstream activation of these two mutations over the whole parameter range (Fig. 4c and the supplement). The rate constant for GAP-stimulated hydrolysis of Gα_q/11_-bound GTP by PLCβ (denoted by *k*_*hyd2*_) was determined to be the most sensitive parameter with respect to the difference in PLCβ activation between the two mutants, while the total concentration of TRIO was the most sensitive parameter with respect to TRIO activation. As the biochemical parameters of this system have not been fully measured, this sensitivity analysis is evaluating the scope of potential behaviors that are within the realm of possibility of available knowledge as much as it is evaluating the robustness of the model.

Although the prevailing assumption is that *GNAQ/11* and *CYSLTR2* mutant networks are effectively equivalent, we were intrigued that our model suggested that the available data do not require them to be equivalent (Fig. 4d). The unknown parameters of the system could potentially allow *CYSLTR2* mutant UM to have strong ERK but weak YAP signaling (~5% of parameter sets sampled), to have weak ERK but strong YAP signaling ( ~ 50% or parameter sets sampled), or to have weak activation of both pathways (~25% of parameter sets sampled). While it is difficult to translate the relative frequency of behaviors over the entire parameter range into predictions about the true parameter values and behavior, we were intrigued at the possibility of there being a lack of functional equivalency between the *GNAQ/11* and *CYSLTR2* mutants as this is counterintuitive based on the canonical view of the pathway. As recent studies have highlighted both ERK and YAP signaling as being critical to *GNAQ/11* mutant UM, we thought it was important to experimentally determine whether CysLT_2_R L129Q can also activate both pathways.

### Experiments reveal that while oncogenic *GNAQ* can activate ERK and YAP signaling, oncogenic *CYSLTR2* can only strongly activate ERK

**Fig. 5:**
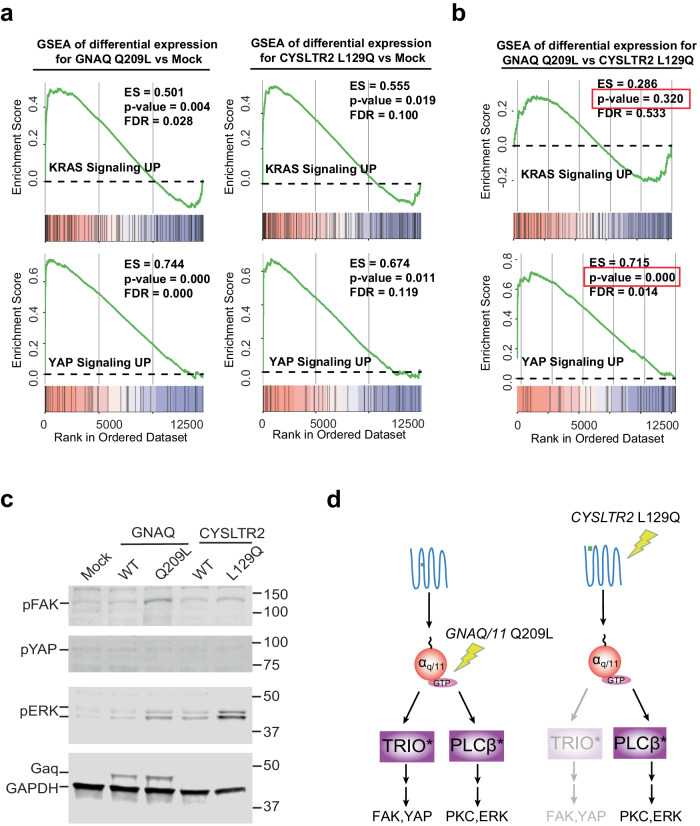
Experimental determination that oncogenic *CYSLTR2* is impaired at activating the FAK/YAP/TAZ pathway. **a** Gene set enrichment analysis (GSEA) of differentially expressed genes for *GNAQ* Q209L and *CYSLTR2* L129Q transfected 293T cells, each compared with mock transfected cells. **b** GSEA of differentially expressed genes between the *GNAQ* Q209L and *CYSLTR2* L129Q transfected conditions for the same cells as in (**a**). **c** Total cell lysates were probed by western blot for phospho-ERK, phospho-YAP, phospho-FAK, Gα_q,_ and GAPDH. Blots are representative of *n* = 3 experimental replicates. **d** Schematic of the experimentally observed differences in the relative strength of downstream activation for the Gα_q_ Q209L and CysLT_2_R L129Q mutations, which identifies which of the two possible diverging behaviors described in Fig. 4d is experimentally demonstrated to occur here. The schematized G-protein alpha subunits (red circles) can represent either (or both) wild-type and mutant forms.

To experimentally explore model-suggested potential for divergent cellular phenotypes, we expressed WT or mutant *GNAQ* or *CYSLTR2* constructs in HEK 293 T cells and quantified downstream signaling and activation of ERK and YAP signaling via RNA-sequencing. HEK 293 T cells have often been used to explore signaling from these mutants^48,49,61,67^ and provide a convenient system for comparing signaling phenotypes of the mutants. (Note: UM cell lines that allow comparisons between *GNAQ/GNA11* and *CYSLTR2* mutants in an isogenic background are not currently available). Gene-set enrichment analysis (GSEA)^68,69^ revealed that both mutants yielded a statistically significant enrichment in the hallmark “KRAS signaling up” signature (active KRAS activates ERK) and the “YAP conserved” signature^70^ compared to the mock transfection control (Fig. 5a, Supplementary Fig. 1). In addition, when the *GNAQ* Q209L expressing cells’ gene expression signature was compared to the gene expression signature of the cells expressing *CYSLTR2* L129Q, it was noted to be significantly enriched in the “YAP conserved” signature (suggesting that there was much more YAP signaling in the *GNAQ* Q209L cells) while there was no enrichment in the hallmark “KRAS signaling up” signature (suggesting that both *GNAQ* and *CYSLTR2* mutants activated KRAS/ERK to more similar levels) (Fig. 5b). We validated these signaling differences by western blot (Fig. 5c). Based on these experiments, the *CYSLTR2 L129Q* mutant appears to have strong ERK activation but weak YAP signaling, in contrast to the *GNAQ* Q209L mutant, which seems to activate both pathways strongly (Fig. 5d).

### Iterative evaluation and adjustment of model parameters based on the new experimental data

**Fig. 6:**
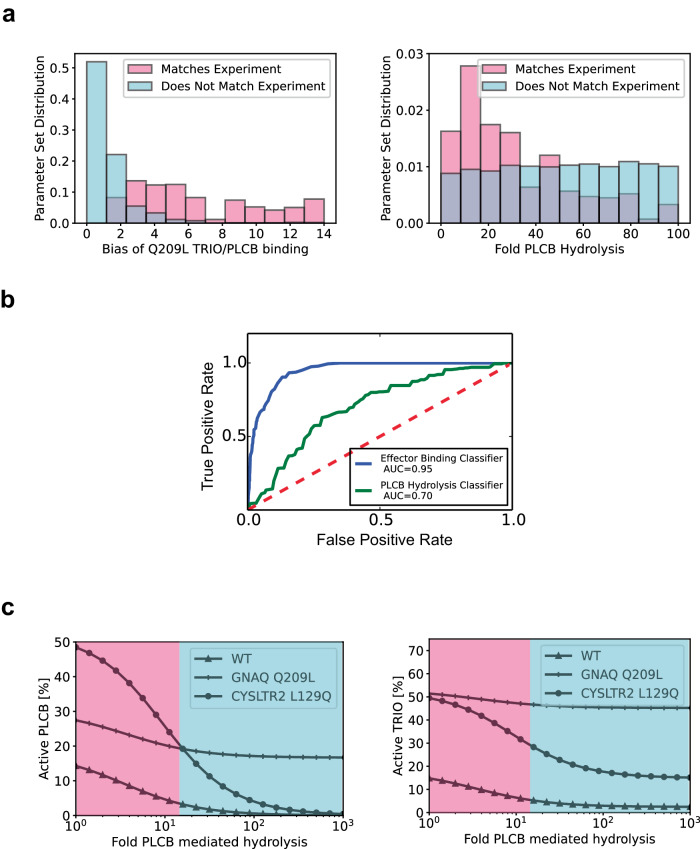
Experimentally informed re-evaluation of parameter sets and parameter ranges. **a** Normalized distributions of parameter values: the bias of the Gα_q_ Q209L mutant binding effectors (denoted by *k*_*at2*_^*mut*^*/k*_*at1*_^*mut*^) and the GAP-stimulated hydrolysis of Gα_q_-bound GTP by PLCβ (denoted here as the fold over basal hydrolysis *k*_*hyd2*_*/k*_*hyd*_) for simulations that qualitatively reproduced the experimental activation patterns (shown in pink) and simulations that did not (shown in cyan). A total of *n* = 11,200 parameter sets were simulated (Fig. 4c and the supplement). **b** Plotted are the receiver operating characteristic (ROC) curves for using either: (1) the bias of the Gα_q_ Q209L mutant binding effectors (denoted by *k*_*at2*_^*mut/wt*^*/k*_*at1*_^*mu/wt*^) with a threshold between *k*_*at2*_^*mut/wt*^*/k*_*at1*_^*mu/wt*^ ≥1 and *k*_*at2*_^*mut/wt*^*/k*_*at1*_^*mu/wt*^ ≥14, or (2) the rate constant for GAP-stimulated hydrolysis of Gα_q_-bound GTP by PLCβ (denoted here as the fold over basal hydrolysis *k*_*hyd2*_*/k*_*hyd*_) with a threshold between *k*_*hyd2*_*/k*_*hyd*_ <1 and *k*_*hyd2*_*/k*_*hyd*_ <100, as a binary classifier to identify whether the model simulated at various parameter sets either matched or did not match the experiment. It is worth noting that *k*_*hyd2*_*/k*_*hyd*_ was set to roughly 770 in Fig. 2 based on the available data. **c** Active TRIO and PLCβ levels resulting from simulation of the mathematical model for the WT, *GNAQ* Q209L, and *CYSLTR2* L129Q settings over a range of GAP-stimulated hydrolysis of Gα_q_-bound GTP by PLCβ (denoted here as the fold over basal hydrolysis *k*_*hyd2*_*/k*_*hyd*_). The pink region denotes the parameter range for which the simulations qualitatively reproduced the experimental activation patterns and the cyan region denotes the parameter range for which the simulations did not qualitatively reproduce the experimental activation patterns. Based on the experimental observations in Fig. 5 and the analysis in (**a**, **b**) above, the bias of Q209L TRIO/PLCB binding was set to *k*_*at2*_^*mut/wt*^*/k*_*at1*_^*mu/wt*^ = *4* for these simulations.

We determined which simulated alternative parameter sets matched our experimentally observed activation patterns and which did not (i.e., which parameter sets gave the result that the CysLT_2_R L129Q mutation yielded greater than or equal levels of PLCβ activation and lower levels of TRIO activation compared to the Gα_q_ Q209L mutation). The two parameters which showed the most significant discrimination between which simulations matched the experiments and which simulations did not match the experiments based on a Kolmogorov-Smirnov test were: the bias of the Gα_q_ Q209L mutant binding effectors to PLCβ relative to TRIO (denoted by the ratio of the association rate constants for each reaction: *k*_*at2*_^*mut/wt*^*/k*_*at1*_^*mut/wt*^) and the rate constant for GAP-stimulated hydrolysis of Gα_q_-bound GTP by PLCβ (*k*_*hyd2*_) (Fig. 6a, Supplementary Fig. 2 and the supplement). A receiver operating characteristic (ROC) analysis suggested that, for the given parameter ranges, *k*_*at2*_^*mut/wt*^*/k*_*at1*_^*mut/wt*^ was the more effective binary classifier for which simulations matched the experiments and which simulations did not match the experiments (Fig. 6b). Of note, co-immunoprecipitation studies that compared the abilities of Gα_q_ Q209L and Gα_q_ WT to bind to TRIO and PLCβ suggested a modest bias for TRIO over PLCβ effector binding for Gα_q_ Q209L compared to Gα_q_ WT^44^. The rate constant for GAP-stimulated hydrolysis of Gα_q_-bound GTP by PLCβ (*k*_*hyd2*_) was another critical parameter. Parameter sets in which *k*_*hyd2*_ was ~1–40-fold faster than basal hydrolysis were more likely to match the experimental results than parameter sets in which *k*_*hyd2*_ was >50-fold faster than basal hydrolysis (Fig. 6a).

To explicitly compare the behavior of the Gα_q_ Q209L and CysLT_2_R L129Q mutations at varying values of *k*_*hyd2*_, we plotted the levels of active PLCβ and active TRIO for each (Fig. 6c), while keeping other parameters fixed. This suggests that when PLCβ has very strong GAP activity (i.e., rapidly promotes the conversion of GTP-bound Gα subunits to GDP-bound Gα subunits), our model of the CysLT_2_R L129Q mutation will not yield significant steady-state activation of PLCβ because all of the Gα subunits are WT and thus fully GAP sensitive (i.e., any Gα_q_-bound GTP will be quickly hydrolyzed by PLCβ resulting in termination of the signal). Overall, these experimental and computational analyses suggest that there is a significant difference in the downstream activation patterns of activating *GNAQ* and *CYSLTR2* mutations. Specifically, the *CYSLTR2* L129Q mutant appears to be relatively deficient in activating the TRIO- > FAK, YAP pathway compared to the *GNAQ* Q209L mutant.

We also present an updated set of parameter estimates that is similar to our initial parameters, but where we have chosen new values for parameters *k*_*at2*_^*mut/wt*^*/k*_*at1*_^*mut/wt*^ and *k*_*hyd2*_ based on this analysis (see the supplement for details). These new parameters are able to reproduce all of the same behaviors as our initial parameter set and they also better reproduce the qualitative differences in downstream signaling observed during our new experiments with *CYSLTR2* and *GNAQ* mutants (Supplementary Fig. 3). We therefore suggest these updated parameters, combined with the model, may serve as a good foundation for further model-driven studies of UM.

### Bioinformatic analysis of patient data reveals *CYSLTR2* mutations co-occur with semaphorin/plexin gene mutations

**Fig. 7:**
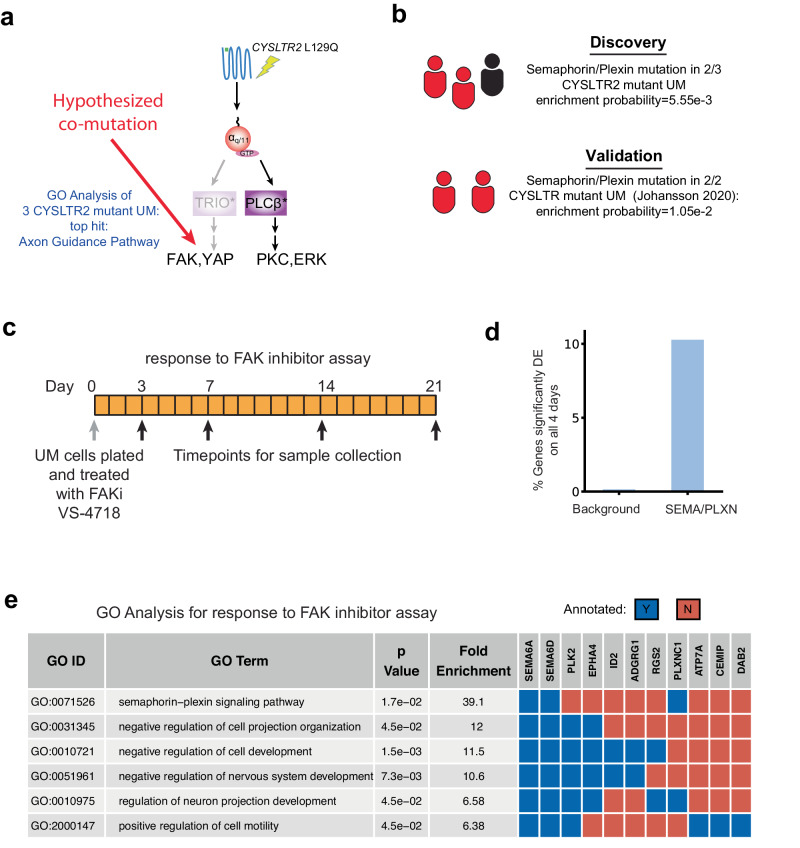
Bioinformatic analysis suggest a role of the semaphorin/plexin signaling network in uveal melanoma. **a** With CysLT_2_R L129Q deficient at activating FAK/YAP/TAZ signaling relative to Gα_q_ Q209L, we hypothesize that there may be co-occurring genetic and/or epigenetic changes that contribute to FAK/YAP/TAZ activation. The schematized G-protein alpha subunits (red circles) represent the wild-type form. Gene Ontology (GO) analysis of the mutations observed in the four published, exome sequenced, *CYSLTR2* L129Q UM^53,54^ identifies the Axon Guidance Pathway as the most enriched pathway. **b** Schematic highlighting 2 of the 3 patients with *CYSLTR2* L129Q mutation in the TCGA have a semaphorin/plexin co-mutation, which was done in our discovery phase. We then investigated a separate Uveal Melanoma data set (ref. ^82^) and found two of the two *CYSLTR2* L129Q patients had a semaphorin or plexin gene mutated. **c** Schematic of assay to treat UM 92.1 cells with the selective FAK inhibitor VS-4718 and collect samples for bulk RNA-sequencing. **d** Percentage of genes that were significantly up or downregulated on all 4 days of sample collection genome-wide vs. the semaphorin/plexin pathway. **e** Gene Ontology (GO) analysis of the 36 genes that were significantly up or downregulated on all 4 days of sample collection.

Based on our computational and experimental analysis presented in the previous sections, we propose the hypothesis that CysLT_2_R L129Q mutations may be deficient in TRIO- > FAK, YAP pathway activation compared to Gα_q_ Q209L mutations. This hypothesis raises the important question: what is happening in UM patients with *CYSLTR2* L129Q mutations? While there are many potential explanations, one intriguing possibility is that there are compensating mutations in UM patients with *CYSLTR2* L129Q mutations that provide additional FAK- > YAP pathway activation (Fig. 7a). None of the UM genomics studies have reported such co-occurring mutations, but the limited number of *CYSLTR2* mutant UM (less than ten cases sequenced to date) leaves this subset underpowered for unbiased discovery analysis of the mutation data. Of note, in a previous bioinformatics analysis of TCGA data, *CYSLTR2* mutant UM patients do not localize to specific molecular or clinical subsets of UM^43^, suggesting there is ultimately similar signaling activity in these patient tumors. We also performed a differential expression analysis of *GNAQ/GNA11* mutant vs. *CYSLTR2* mutant patients in TCGA followed by GSEA, which revealed no statistically significant difference in either of the “KRAS signaling up” or “YAP conserved” signatures mentioned previously (Supplementary Fig. 4a). It is worth noting that of the 14 genes identified as significantly differentially expressed between *GNAQ/GNA11* and *CYSLTR2* mutant UM patient samples in TCGA, *PRAME* (preferentially expressed in melanoma) has previously been identified as independent marker of metastasis in UM^71^ (Supplementary Fig. 4b).

Although the number of genomically sequenced *CYSLTR2* mutant UM samples may be underpowered for unbiased discovery analyses, we speculated that the number may be sufficient for focused exploration of genes that could potentially activate FAK- > YAP signaling. We first analyzed UM patient data in TCGA using cBioPortal^53,54^. A gene ontology (GO) analysis^72–74^ of the list of genes mutated in these *CYSLTR2* mutant patients using the Reactome Knowledgebase^75^ yielded a statistically significant enrichment of the axon guidance pathway (Fig. 7a, Supplementary Fig. 4c). Inspection of the genes revealed a statistically significant enrichment of mutations in the semaphorin/plexin signaling family in UM patients with *CYSLTR2* L129Q mutations (Fig. 7b). Semaphorins are membrane-bound or diffusible ligands that signal through the plexin receptors and were originally identified as important in axon guidance and angiogenesis but have more recently been implicated in several aspects of cell-cell communication, cancer^76^ and FAK activation^77–79^. Previous work has identified that BAP1 loss in UM can lead to significant deregulation of axon guidance pathways, including several semaphorin/plexin genes^80^ and another study identified several semaphorin genes as differentially expressed in melanocytes expressing GNAQ Q209L^81^. Thus, there were some reasons to speculate that the observed mutations in the semaphorin/plexin pathway may be providing the hypothesized contribution to FAK activation. Importantly, UM is relatively unique among malignancies that impact adults in that there are typically a very low number of coding mutations. Thus, there were few co-occurring mutations within the *CYSLTR2* mutant cancers (between 5 and 19 coding mutations per sample), intuitively reducing the chance that the enrichment of semaphorin/plexin mutations in *CYSLTR2* mutant UM relative to *GNAQ*/*GNA11* mutant UM was a false discovery. Our analysis of co-occurring *BAP1* monosomy or mutation as a covariate found no significant correlation with *CYSLTR2* mutations and *BAP1* monosomy/mutation.

To prospectively test whether this observed enrichment of mutations existed in other available datasets, we found and analyzed additional UM genomics data^82^. Of note, these data included two additional patients with *CYSLTR2* L129Q mutations and both samples have coding mutations in the semaphorin/plexin pathway (Fig. 7b). Thus, a total of 4/5 patients with *CYSLTR2* L129Q mutations from TCGA and ref. ^82^ have at least one co-occurring mutation in the semaphorin/plexin signaling family. Analysis of the observed semaphorin/plexin mutations observed in TCGA and ref. ^82^ with PolyPhen2^83^ revealed two of the mutations (*PLXNA4* T642I and *PLXNA4* R1626Q) were predicted to be damaging while two mutations (*PLXND1* Q657H and *SEMA7A* E183K) were predicted to be benign. The observed mutations in *PLXNA4* and *PLXND1* in TCGA are found in similar domains of the respective proteins (Supplementary Fig. 4d). We also observed a statistically significant difference in disease-specific survival in UM patients with low expression of *PLXNB1* and *PLXNA1* mRNA and high expression of *PLXNC1* and *SEMA4D* mRNA, thus providing additional circumstantial evidence that plexin/semaphorin signaling has a role in uveal melanoma (Supplementary Fig. 5a). There are several reports in the literature of the tumor suppressor effects of semaphorin/plexin signaling in cutaneous melanoma^84–86^. Thus, although a role for semaphorin/plexin signaling in uveal melanomagenesis has only been briefly mentioned in the literature^80,81^, our analysis finds a variety of suggestive data.

### Treatment of GNAQ mutant uveal melanoma cells with FAK inhibitors further suggests a role for semaphorin/plexin signaling in UM

FAK inhibitors are being evaluated for use in UM^50,87^. We hypothesized that the semaphorin/plexin pathway may play a role in the response to FAKi targeted therapy in the UM context. To experimentally test this hypothesis, we continually passaged *GNAQ* mutant UM 92.1 cells in either 100 nM of the selective FAK inhibitor VS-4718 or DMSO control for 21 days (Fig. 7c). At this dose, there is a strong suppression of signaling (Supplementary Fig. 5b) but little to no suppression of proliferation (Supplementary Fig. 5c). We hypothesized that there may be compensatory changes in semaphorin and/or plexin genes that counteract the targeting of FAK to maintain proliferation at these modest doses of FAK inhibitor. To evaluate, we performed RNA-sequencing on samples collected at 0, 3, 7, 14, and 21 days. Differential expression analysis revealed that several of the semaphorin/plexin genes were significantly up or downregulated in response to continued FAK inhibitor treatment compared to DMSO control, with 3/29 or 10.34% of these genes significantly upregulated on all 4 days of sample collection (Fig. 7d). In comparison, the background rate of genes significantly up or downregulated on all 4 days of sample collection was 0.21%. A GO analysis of the 36 genes that were significantly up or downregulated on all 4 days of sample collection revealed that the semaphorin-plexin signaling pathway was the most significantly enriched GO term and that all of the GO terms that were significantly enriched contained *SEMA6A* and *SEMA6D* (Fig. 7e). The patterns of expression for each semaphorin/plexin gene in response to FAK inhibitor treatment can be seen in Supplementary Fig. 5d.

## Discussion

In this study, we investigated whether biochemical-mechanism based mathematical models of critical oncogenic signaling networks could be used to help open new areas for research into mechanisms of disease.

We here found this to be the case: the model-based analysis first revealed that pathogenic and mutually exclusive *GNAQ/11* and *CYSLTR2* mutations are not functionally equivalent. We found that although the dual-activation of ERK and YAP pathways that is essential for UM can be generated by a canonical *GNAQ* mutation, a pathogenic *CYSTLR2* mutation is only able to strongly activate the ERK pathway and may require an additional, co-occurring event to activate the YAP pathway. The lack of equivalency was first suggested by our mathematical modeling, tested and confirmed experimentally, and then evaluated with genomic and transcriptomic analyses. This new understanding uncovered the potential mechanism that *CYSLTR2* mutant UM use to activate the YAP pathway: deregulation of the semaphorin/plexin pathway.

There are reports of various semaphorin/plexin mutations in the context of cutaneous melanoma and other cancers^88,89^. However, a role for semaphorin and plexin signaling in UM does not have extensive prior evidence. Our mathematical modeling led us to uncover signaling differences between oncogenic Gα_q_ and oncogenic CysLT_2_R, which we then experimentally confirmed. We more generally hypothesize that there may be signaling differences for *PLCB4* mutant melanoma and for other mutant forms of *GNAQ/11* and of *CYSLTR2*. Additional characterization of signaling by other mutants found in UM appears to be an important area for more experimental investigation that our model-based study has motivated.

Our hypothesis that secondary, compensating mutations are required for *CYSLTR2* mutant UM provides a potential explanation for the relative scarcity of UM patients with *CYSLTR2* L129Q mutations. If both Gα_q/11_ and CysLT_2_R mutations behaved exactly the same in terms of signaling activation, they might be expected to occur in more even proportion in UM patients. On the other hand, if *CYSLTR2* L129Q mutant tumors need a second compensating mutation to become fully oncogenic, it is logical that they would be rarer. An important test of our hypothesis in the future will be if the enrichment of semaphorin/plexin mutations we identified for *CYSLTR2* mutant UM patients is confirmed as more patient samples are sequenced. Of note, there was one additional UM patient sample^90^ that harbors a *CYSLTR2* L129Q mutation and that is not in either of the datasets mentioned previously. This patient was identified to have a *PLXND1* coding mutation^90^, but upon our re-analysis of the raw data we determined that the mutation was intronic. While we cannot easily determine the meaning of this mutation, it is at the very least an intriguing coincidence, and could potentially be functional^91,92^. Another area of future exploration suggested by this model-driven study is additional sequencing of UM patients, specifically to evaluate co-occurrence of plexin and semaphorin gene mutations. Biochemical and biophysical characterization of plexin and semaphorin gene mutations is another emerging area for future study, as motivated by this study.

The enrichment of plexin and semaphorin mutations in *CYSLTR2* mutant UM patients and evidence of survival differences based on gene expression make a strong case that plexin and semaphorin proteins play a role in uveal melanoma. As this is a large family with related, but diverging, roles, a more thorough characterization of the different plexins and semaphorin proteins in uveal melanoma, including context-specific function, cross-talk, and feedback would provide important information that is needed to more fully interpret experimental data and could potentially identify new therapeutic strategies.

Our computational analyses reveal that the effector binding properties of the Gα_q_ mutant and the rate constant for GAP-stimulated hydrolysis of Gα_q_-bound GTP by PLCβ are critical for our mathematical model to reproduce the experimentally observed activation patterns. This result was not intuitive to experimental biologists working in the field or to computational biologists working on the model. That the behavior of the system depends upon the specific parameters of the model further highlights that it is not possible to simply look at a model schematic and infer model outcomes. However, once an empirical observation is made and parameters of the model are found that reproduce the empirical observations, the model may facilitate the development of an intuitive understanding of the system behavior. In this study, we experimentally found that CysLT_2_R L129Q is impaired at activating TRIO/YAP signaling relative to Gα_q/11_ and does not seem to be impaired at activating PLCβ/ERK. This was the opposite of the computational inference that resulted from using our initial parameter estimates.

Our revised parameter estimates are consistent with the experimental observations and consideration of how parameters needed to change to match the observations does help produce an intuitive understanding of the system. The observed behavior of the two mutants, and the parameter changes, may be intuitively understood as follows: (a) our initial estimate for PLCβ-mediated hydrolysis of active, GTP-bound, WT Gα_q/11_ was too large, resulting in a profound inhibition of PLCβ (and thereby ERK) activation even when CysLT_2_R L129Q was driving WT Gα_q/11_ activation. Correspondingly, alternative parameter sets where the value of PLCβ-mediated hydrolysis of active, GTP-bound, WT Gα_q/11_ was smaller than the original estimate were more likely to result in activation of PLCβ/ERK. The other parameters that needed to be updated for the model to be more consistent with empirical observations involved the association rate constants between mutant Gα_q/11_ with its two effectors, TRIO and PLCβ. If Gα_q/11_ Q209L has similar affinity for TRIO as WT Gα_q/11_, and if both G_αq/11_ Q209L and CysLT2R L129Q induce similar total levels of GTP-bound G_αq/11_ (i.e., WT and mutant combined), then one would expect G_αq/11_ Q209L and CysLT_2_R L129Q to bind TRIO and activate YAP/TAZ signaling similarly. However, the experiments found that CysLT_2_R L129Q was impaired at activating YAP/TAZ signaling. Our updated parameters therefore assume an elevated association rate constant for G_αq/11_ Q209L binding to TRIO so that the model better matches the experimental observation.

Our finding that CysLT_2_R L129Q is not impaired at activating ERK suggests that the PLCβ isoforms present in uveal melanoma catalyze hydrolysis to a more modest extent. A consideration of experimental reports of these parameters can help evaluate the potential validity of this inference. For our initial parameterization, we chose the rate constant for GAP mediated hydrolysis by PLCβ based on one study that reported the rate constant for GAP mediated hydrolysis of PLCβ was up to 1000-fold faster than basal hydrolysis for PLCβ1^51^. Other isoforms in other studies are reported to have a wide range of rate constants^93^. The rate constant for GAP mediated hydrolysis of PLCβ in our model can be thought of as an aggregate effect from all the PLCβ isoforms present. It should also be noted that the binding of Gα_q_ to PLCβ has been reported to exhibit anomalous affinity^94^ and there may be more complex activation mechanisms that are beyond the scope of our current model.

We highlight several important qualifiers for our parameter sets. Our initial parameter estimates represented reasonable first estimates based on the literature, such as what one would use when initiating a new study. Our revised parameter estimates are an updated set that is more consistent with the experimental observations, but is not formally fit or optimized. If and when parameters are formally fit to a model, it is common for there to be many alternative parameter sets that can reproduce the behavior of a system^95^. We could therefore have potentially identified a completely different alternative parameter set that would have been effectively equally consistent with the behaviors we would like the model to match. Therefore, our updated parameters should not be interpreted as improved estimates for the underlying biochemical parameter. As another way to state this, in our computational analysis we kept the equilibrium dissociation constants for protein-protein interactions constant and only allowed the association rate constant to vary. Had we instead varied the dissociation rate constant while keeping the equilibrium dissociation constant fixed, we would have estimated changes to dissociation rates that would yield the same overall change in equilibrium dissociation constant. Overall, many other alternative parameterizations could have been found. Future studies could attempt to better constrain parameters, and these overall results highlight a thorough biochemical characterization of PLCβ as an important area for future research in the study of UM molecular pathogenesis. Additionally, the empirical observation of CysLT_2_R L129Q is not impaired at activating ERK but is relatively impaired at FAK activation further suggests that Gα_q/11_ has a bias for binding to PLCβ relative to TRIO; this is another model-based inference that can be pursued in subsequent experimental work.

Lastly, it should also be noted that there are several possible future extensions to the model that could complement any future experimental studies. As an example, explicitly including components of the semaphorin/plexin pathway in the model could help explore possible mechanisms of co-activation with the CysLT_2_R L129Q mutant receptor. Although several aspects of semaphorin and plexin signaling have been investigated with computational and mathematical models^96–100^ biochemical-mechanism based mathematical models do not appear to have heavily utilized with respect to investigations of signaling from plexins through its downstream effectors. Mathematical models that investigate cross-talk between Gα_q/11_ and semaphorin/plexin signaling could potentially be enlightening; for example, a simple model may help estimate how much plexin-driven FAK activation would be needed in combination with a CysLT_2_R L129Q mutant to achieve a total signal comparable to what a Gα_q_ Q209L would induce. We envision this as an area where mathematical modeling could be particularly useful. Other potential modeling directions include explicitly modeling downstream from PLCβ and TRIO, as well as expanding the characterization of PLCβ to investigate pathogenic PLCβ mutations.

One challenge in the development of a mechanistic model is the identification of the appropriate parameter values. For example, even though GPCR and G-protein signaling has been extensively studied, the characterization of fundamental rate constants for reactions and for mutants is an ongoing area of research^44,101–104^. Thus, published mechanistic models that focus on GPCR signaling and downstream G-proteins must estimate and approximate parameter values. In contrast, the RAS GTPases (HRAS/NRAS/KRAS) have been very well studied at the level of component reactions, including for several oncogenic mutations^14,15,105^. This has enabled the development of mathematical models of RAS signaling that can directly incorporate these experimental data as parameters^7,106,107^. These models of RAS have been useful for understanding why pathogenic mutations are activating, for inferring new aspects of cancer genetics, and for understanding mutation-specific treatment responses. However, it has been unclear whether biochemically detailed mechanistic models can also add a meaningful contribution to studies of pathogenic mutations when the chemical-kinetic properties of the pathway and mutations are far less thoroughly quantified. Our work here demonstrates that this approach can still catalyze progress and uncover previously unknown features of a system. It is worth noting that mutations in the *CYSLTR2*/*GNAQ*/*GNA11*/*PLCB4* signaling pathway have been identified in other malignancies such as blue nevi^108,109^ and tumors of the central nervous system^110–113^, and somatic *GNAQ* mutations are known to cause the congenital disorder Sturge-Weber syndrome^67^. Looking forward, our mathematical model could be applicable to these cases as well.

**Fig. 8:**
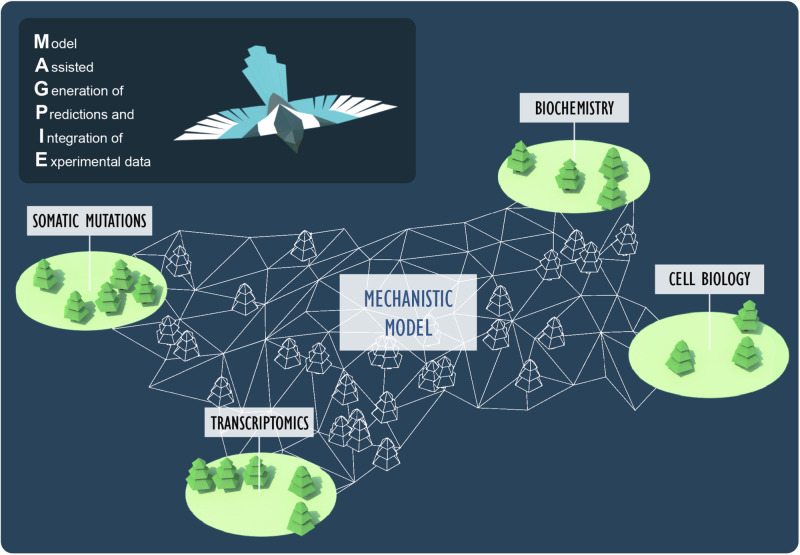
MAGPIE—Model-assisted generation of predictions and integration of experimental data. This work demonstrates how an integrated approach involving mechanistic modeling, bioinformatic analysis, and experimental approaches can uncover new directions and aid in our understanding of cancer biology. The figure was created by illustrator Amy Cao with express permission to use.

This study highlights how model-based inference can help break new ground through the re-analysis of existing data to suggest new ideas that can be tested with focused, hypothesis-driven, experiments. We refer to this approach as MAGPIE (Model Assisted Generation of Predictions and Interpretation of Experimental data) (Fig. 8). The value of this approach can be better understood if one considers why some of these observations were not previously made. For example, although our analysis relies upon readily available genomic data, previous analyses of these data did not identify the co-occurrence of semaphorin/plexin pathway mutations in *CYSLTR2* mutant UM^43,114^. This is likely because a relatively small number of UM patient samples have received exome-level sequencing; only 106 cases are currently listed in cBioPortal. Of those, 97 have *GNAQ*/*GNA11* mutations and nine do not. This is an extremely small dataset, and a non-focused analysis that considers all pathways is unlikely to yield meaningful results. Our mechanistic mathematical modeling led to experimental discoveries, which in turn suggested very focused analyses of the limited genomic data. In this manner, we were able to uncover a new aspect of UM biology that was featured in the available data (biochemical, biophysical, and genomic) but that may not have been detected without an integrated, mechanistic approach to evaluate the pathway. It should be noted that there are several prominent examples in the literature utilizing the combination of mechanistic modeling and data-driven approaches to generate insight into diverse biological systems^115,116^.

Our work highlights the power of integrating multiple computational and bioinformatics methods with experimental and genomic biology. Such integrated studies that iteratively alternate between (new or existing) experimental data and mathematical and computational analyses have great potential for elucidating unknown aspects of cancer biology. For example, the integration of additional datasets to generate novel, evidence-based, hypotheses can allow for focused queries of genomic data where the signal of a biologically important process can be better uncovered than through an unbiased, all-hypotheses considered query of genomic data. Our ability to extract new insights into UM, a rare cancer with a small number of cases that have been sequenced, by focusing on an even smaller number of cases attests to the power of these integrated approaches as a tool for moving forward when vast datasets are not available and mechanism agnostic big data approaches cannot be utilized.

## Methods

### Mathematical model

A description of the mechanistic mathematical model of oncogenic Gα signaling presented here can be found in the main text and the explicit reactions, rate constants, protein abundances, and relevant references can be found in the supplement. Our model is a set of ODEs that we solve numerically using Python (RRID:SCR_008394). The global sensitivity analysis presented in Fig. 4c of the main text was performed using the SALib Python library^117^. Code that allows all computational results and figures to be reproduced is provided in the form of a Python notebook at https://github.com/StitesLab/GNAQ_model. A PDF that shows the executed Python notebook with outputs is also included for convenience.

### Cell line models and culture method

HEK 293T cells were grown in DMEM supplemented with fetal bovine serum (FBS) (10%), penicillin (100 U/ml), and streptomycin (100 μg/ml). 92.1 UM cells (RRID:CVCL_8607) were grown in RPMI media containing 10% FBS (Atlanta Biologicals) and 1% penicillin/streptomycin (Corning).

### Expression plasmid transfection

HEK 293T cells were plated in a 6-well plate in DMEM supplemented with 10% FBS, penicillin (100 U/ml), and streptomycin (100 μg/ml) 24 h before transfection. The following day, cells were transfected with expression plasmids with duplex containing 0.25 μg of DNA, 250 μl of optimem, and 10 μl of Lipofectamine 2000 per well. All constructs were provided by the Gutkind lab and mutations were verified by sequencing in the Stites lab. Cells were harvested 24 h after transfection.

### Western blot analysis

Cell lysates were generated using Lysis buffer (Thermo Fisher Scientific, 1862301) containing protease inhibitor cocktail (Cell Signaling Technology) and incubated on ice for 1 h, with brief vortexing every 5 min. The total protein concentration was determined by Pierce Protein assay (Thermo Fisher Scientific). Protein samples were resolved by electrophoresis on 12% SDS–polyacrylamide gels and electrophoretically transferred to polyvinylidene difluoride (PVDF) membranes (Millipore Corporation) for 20 min at 25 V with the trans-blot turbo (Bio-Rad Laboratories). The blots were probed with the appropriate primary antibody and the appropriate fluorophore-conjugated secondary antibody. The protein bands were visualized using the Licor CLx Odyssey imaging station (Licor Biosystems) (RRID:SCR_014579). Comparative changes were measured with Licor Image Studio software from independent experiments. The antibodies used are: anti-phospho-Thr202/Tyr204-ERK1/2 (BioLegend, catalog #675502) (RRID:AB_2565604) (1:1000 dilution); anti-phospho-Ser127-YAP (Cell Signaling Technology, catalog #4911) (RRID:AB_2218913) (1:1000 dilution); anti-phospho-Tyr397-FAK (Cell Signaling Technology, catalog #8556) (RRID:AB_10891442) (1:1000 dilution);anti-GNAQ (Cell Signaling Technology, catalog #14373) (RRID:AB_2665457) (1:1000 dilution); anti-GAPDH (Santa Cruz Biotechnology, catalog #47724) (RRID:AB_627678). Multiple blots were run in parallel from the same lysates. Uncropped blots are provided as a source data file within the supplementary methods.

### Bioinformatic analysis of somatic mutations in patient samples

To calculate the probability of the observed enrichment of semaphorin/plexin family mutations in TCGA (RRID:SCR_003193) and ref. ^82^ cited in the main text occurring by chance, we explicitly calculated the number of ways of having the observed number of co-mutations divided by the total number of ways of distributing the mutations at random as follows in Eq. 1:

1 $${enrichment\; probability}=\frac{\left(N-k\right)!}{\left(x-k\right)!\left(y-k\right)!\left(N+k-x-y\right)!}\frac{({{N}\atop{k}})}{{({{N}\atop{x}})}{({{N}\atop{y}})}}$$

Where *N* = total number of patients (80 for TCGA, 103 for ref. ^82^), *k* = number of co-mutations (2 for TCGA, 2 for ref. ^82^), *x* = total number of *CYSLTR2* mutations (3 for TCGA, 2 for ref. ^82^) and *y* = total number of semaphorin/plexin mutations (4 for TCGA, 11 for ref. ^82^). We also independently confirmed this expression via Monte Carlo simulation. This value describes the probability of observing this enrichment of mutations by chance for the given number of mutations and patients. All other analysis of patient data presented in Supplementary Fig. 4 was performed using cBioPortal (RRID:SCR_014555)^53,54^.

### Bulk RNA-sequencing analysis

HEK 293T cells were transfected as described above and harvested 36 h after transfection. RNA was isolated using miRNeasy (QIAGEN) and mRNA sequencing libraries were prepared according to manufacturer’s protocol using RNA using Illumina TruSeq Stranded mRNA library preparation kit (Illumina). Three independent biological replicates were obtained for transfections, lysates, and RNA preparation. Raw reads from bulk RNA-sequencing were trimmed with Trim Galore (RRID:SCR_011847) v0.4.4_dev (https://www.bioinformatics.babraham.ac.uk/projects/trim_galore/) and quality-checked with FastQC v0.11.8 (RRID:SCR_014583) (http://www.bioinformatics.babraham.ac.uk/projects/fastqc). Trimmed reads were then aligned to the hg38 human reference genome with STAR aligner v2.5.3a^118^ (RRID:SCR_004463), and converted to gene counts with the analyzeRepeats.pl script in HOMER^119^ (RRID:SCR_010881). Differential expression: Gene counts were normalized and queried for differential expression using DESeq2 v1.30.0^120^ (RRID:SCR_015687). For each pairwise comparison, genes with fewer than 20 total raw counts across all samples were discarded prior to normalization, and genes with an absolute log2foldchange > 1 and an FDR-corrected *p* ≤ 0.05 were pulled as significant. Functional Enrichment: Genes were queried for treatment-specific functional enrichment using over-representation analysis (ORA) and gene set enrichment analysis (GSEA) in WebGestaltR v0.4.4^121^. Differentially expressed genes in each pairwise comparison were queried against the biological process ontology with ORA, while pairwise GSEAs were used to query pathway gene sets from KEGG (RRID:SCR_012773) (https://www.genome.jp/kegg/pathway.html), and MSigDB (http://www.gsea-msigdb.org/gsea/msigdb/collections.jsp).

### Cancer cell drug adaptation assay

92.1 UM cells were treated with either 100 nM of the FAKi VS-4718 (provided by the Gutkind lab) or DMSO for a period of 21 days. Treatments and media were refreshed every 72 h. Cells were plated for each condition in triplicate, and three biological replicates of the complete experiment were performed. RNA was isolated using E.Z.N.A. Total RNA kit I (Omega) at intervals of 0, 3, 7, 14, and 21 days. mRNA sequencing libraries were prepared according to manufacturer’s protocol using RNA using Illumina TruSeq Stranded mRNA library preparation kit (Illumina).

### Supplementary information

Supplementary Material

## Data Availability

All information needed to reproduce the model are included in the Supplementary Information. RNAseq data from the experiments new to this manuscript are available from the Gene Expression Omnibus (GEO) with accession numbers GSE267152 and GSE267153. A Python notebook that includes all of the information needed to reproduce the model is available at https://github.com/StitesLab/GNAQ_model. A PDF version is also included to facilitate review, in case one does not have Python installed.
