# Supplementary material for: Systems modeling of oncogenic G-protein and GPCR signaling reveals unexpected differences in downstream pathway activation
Source: NPJ Syst Biol Appl. 2024 Jul 16;10:75. doi: 10.1038/s41540-024-00400-1 (PMC11252164; doi:10.1038/s41540-024-00400-1)
Supplement: Supplementary file 1 — Supplementary Material [file 41540_2024_400_MOESM1_ESM.pdf]

Systems modeling of oncogenic G-protein and GPCR signaling reveals unexpected differences in downstream pathway activation

Michael Trogdon, Kodye Abbott, Nadia Arang, Kathryn Lande, Navneet Kaur, Melinda Tong, Mathieu Bakhoum, J. Silvio Gutkind, Edward C. Stites

Supplementary Information

- Supplementary Figures 1-5
- Source Data File
- Supporting Information
- Supplementary References

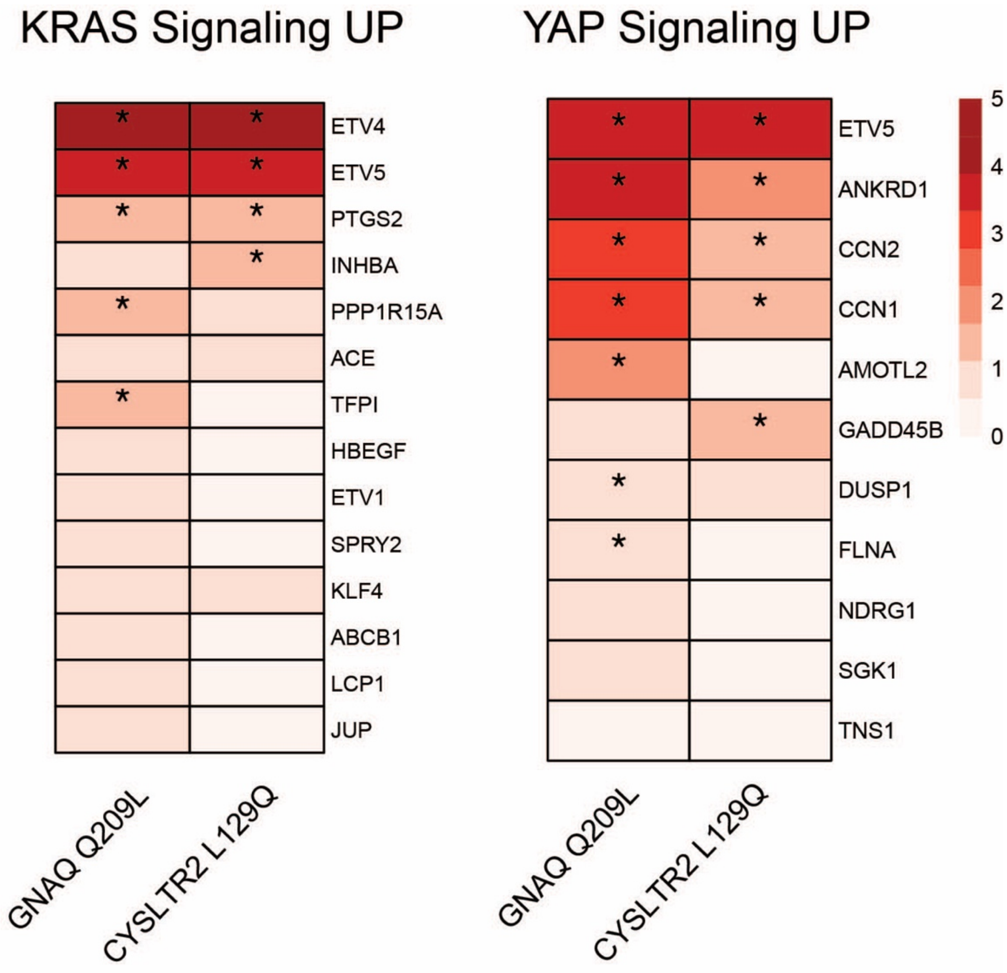

Supplementary Figure 1. Gene expression heatmap from overexpression studies of oncogenic GNAQ and oncogenic CYSLTR2. HEK 293T cells were transfected with either: mock control, *GNAQ* Q209L or *CYSLTR2* L129Q constructs. Samples were prepped for bulk RNA-sequencing. Results are based on n=3 experimental replicates. Gene differential expression heatmap of top overexpressed genes (log2FC >0.5) for each condition compared to the mock control. The colorbar corresponds to the log2FC compared to mock control and the * denotes significance as determined by an FDR-corrected p-value ≤ 0.05.

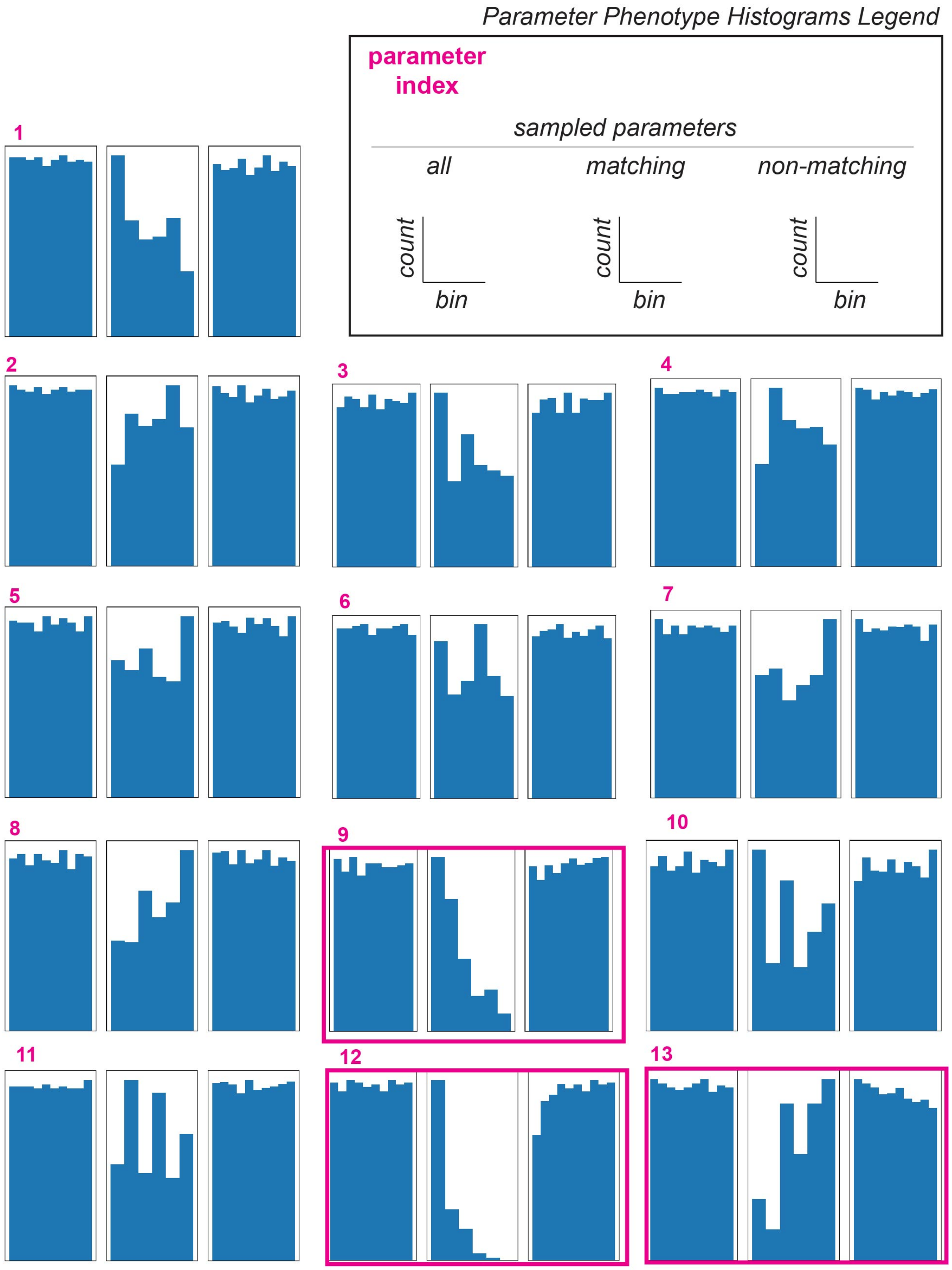

Supplementary Figure 2. Histograms presenting the distribution of parameter values that match or do not match the experimentally observed deficit of *CYSLTR2* L129Q for activating the YAP/TAZ pathway along with the distribution of all the values sampled for each parameter. Parameter values were deemed to match the experiment if simulation of the model resulted in the $CysLT_2R$ WT/L129Q case having greater than or equal PLCβ activation and lower TRIO activation than the $G\alpha_q$ WT/Q209L case; otherwise the parameter set was considered not to match the experiment. The three most significant parameters discussed in the main text are highlighted in pink. The pink parameter indexes correspond to the following parameters, which are further described in the supplement: 1: total amount of heterotrimeric G-protein, 2: basal amount of active receptor, 3: total amount of TRIO, 4: total amount of PLCβ, 5: total amount of RGS, 6: $G\alpha_q$GTP binding PLCβ, 7: $G\alpha_q$GTP binding TRIO, 8: $G\alpha_q$GTP binding RGS, 9: fold PLCβ hydrolysis, 10: fold basal hydrolysis Q209L, 11: GEF stimulation by active receptor, 12: Bias of Q209L binding PLCβ, 13: Bias of Q209L binding TRIO.

a

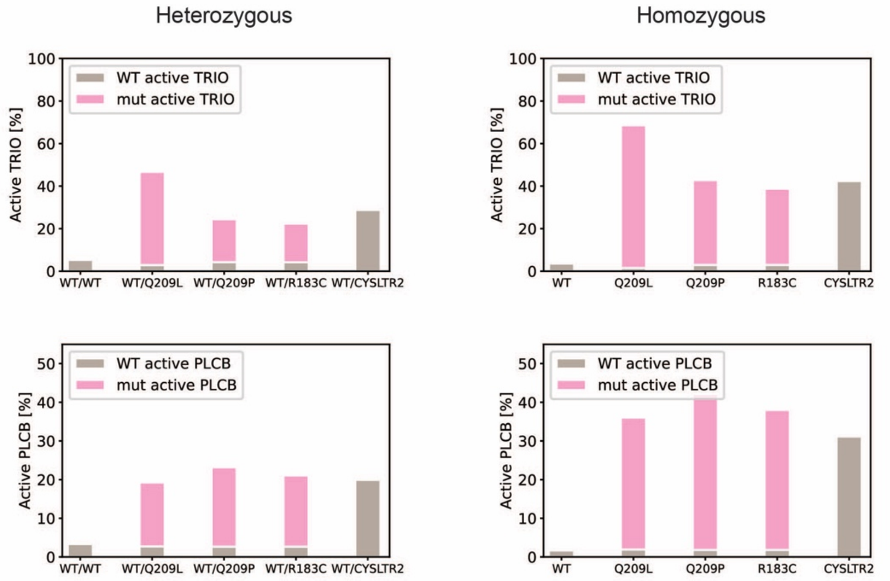

b

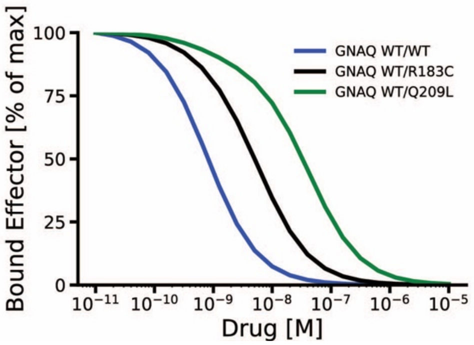

Supplementary Figure 3. Evaluation of refined parameter estimates. (**a**) Characterization of the kinetics-based dynamic equilibrium mathematical model of G$\alpha_{q/11}$ and CysLT$_2$R signaling in UM. Active TRIO and PLCβ levels resulting from simulation of the model for modeled heterozygous (one copy) and homozygous (two copies) of *GNAQ/11* Q209L, and *CYSLTR2* L129Q compared to the modeled all *GNAQ/11* and *CYSLTR2* WT genotype. (**b**) Model simulations of G$\alpha_{q/11}$-inhibitor dose responses on modeled GNAQ/11 WT, heterozygous R183C, and heterozygous Q209L genotypes.

a

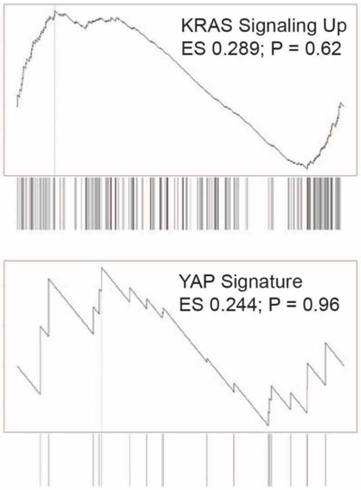

b

Gene	Log2FC	Adjusted p-value
EFCAB1	6.08	0.04
HOXD1	5.98	0.03
GSTM1	5.86	0.04
FLG	3.78	0.01
GALNTL6	5.76	0.05
SLC24A3	3.52	0.02
PRAME	7.83	0.01
ZNF846	2.12	0.01
CTD.3065316.6	-4.11	0.01
HTN3	21.5	2.6e-9
HOXD.AS1	4.81	0.01
PSPHP1	21.4	1.44e-8
AC009336.24	5.87	4e-3
FLG.AS1	4.34	5e-4

c

	Homo sapiens (REF)	upload_1 (▼ Hierarchy NEW! ?)					
Reactome pathways	#	#	expected	Fold Enrichment	+/-	raw P value	FDR
Axon guidance	547	23	8.29	2.78	+	1.72E-05	3.92E-02

d

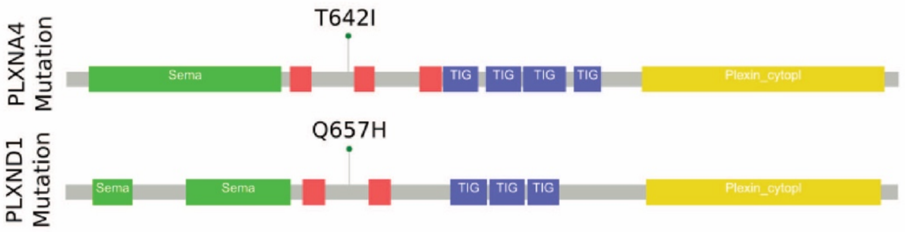

Supplementary Figure 4. Analysis of CYSLTR2 mutant and Plexin/Semaphorin alteration UM. (**a**) GSEA reveals no difference in either the "KRAS signaling up" (FDR=1, p-value=0.62) or the "YAP conserved" (FDR=1, p-value=0.96) signature after performing differential expression analysis of UM patients with *GNAQ/GNA11* mutations vs UM patients with *CYSLTR2* L129Q mutations from TCGA and cBioPortal [53,54]. (**b**) Genes found to be significantly differentially expressed (absolute log2 fold change>0.5 and adjusted p-value<0.05) between the two patient populations (a positive fold change indicates the gene is more highly expressed in GNAQ/GNA11 mutant patients). (**c**) GO enrichment analysis of co-occurring mutations found in *CYSLTR2* mutant UM. (**d**) Location of observed *PLXNA4* and *PLXND1* mutations in CYSLTR2 L129Q mutant UM patients from TCGA and cBioPortal [53,54]

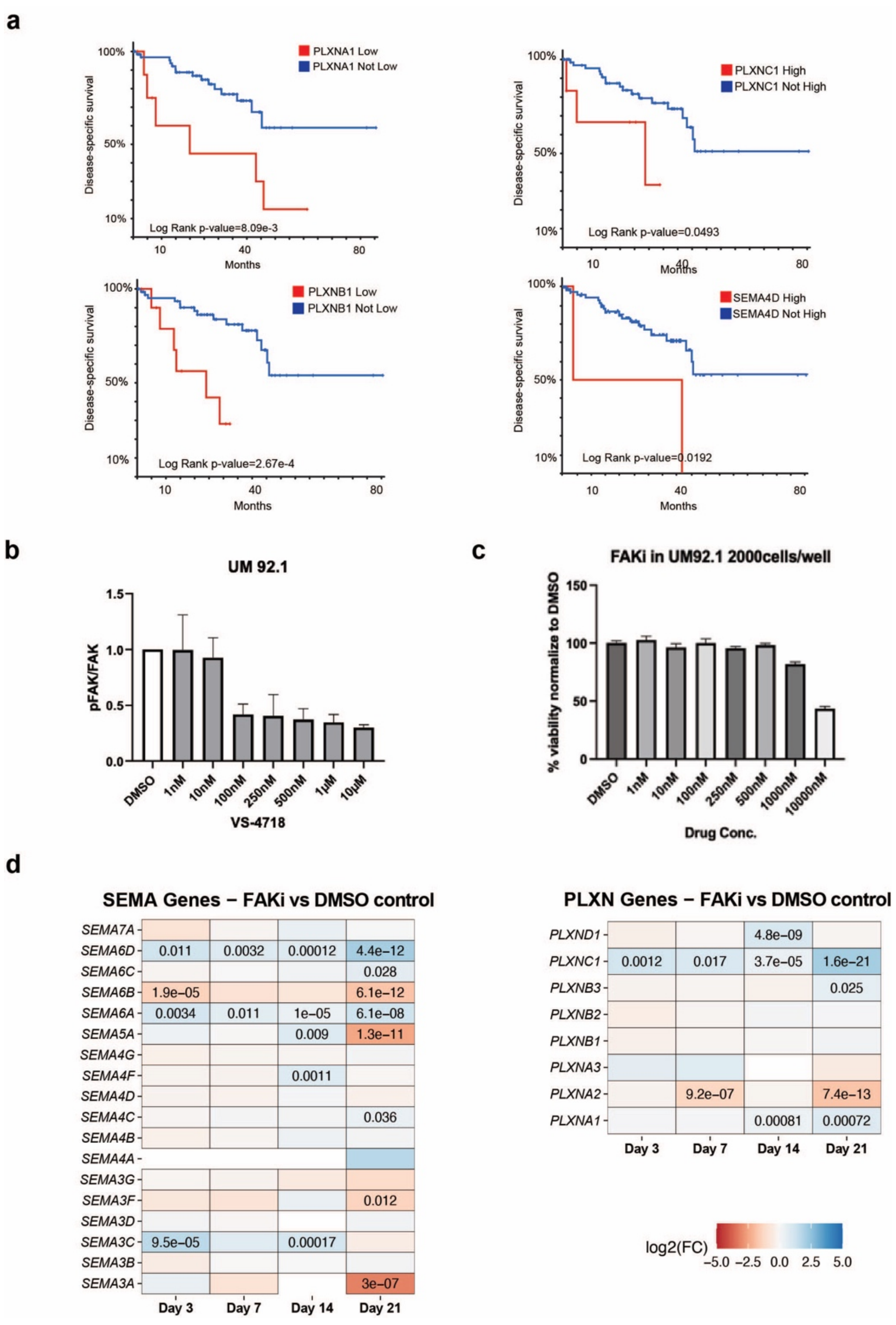

Supplementary Figure 5. Additional evidence that semaphorin and plexin genes impact uveal melanoma phenotypes. (**a**) Disease-specific survival for UM patients with low expression of *PLXNB1* and *PLXNA1* mRNA (z-score<-2) and with high expression of *PLXNC1* and *SEMA4D* (z-score>2) from TCGA and cBioPortal [53,54]. (**b**) Normalized levels of phosphorylated FAK for UM 92.1 cells treated with the indicated dose of FAK inhibitor VS-4718. Error bars indicate standard deviation, n=3. (**c**) Normalized cellular proliferation assays for UM 92.1 cells treated with the indicated dose of FAK inhibitor VS-4718. Error bars indicate standard deviation for 4 technical replicates within this experiment. Results are representative of 3 biological replicates. (**d**) Heatmap of differentially expressed genes in the semaphorin/plexin family for FAK inhibitor treated vs DMSO treated control for each day of sample collection. Entries are annotated with the adjusted p-value<0.05.

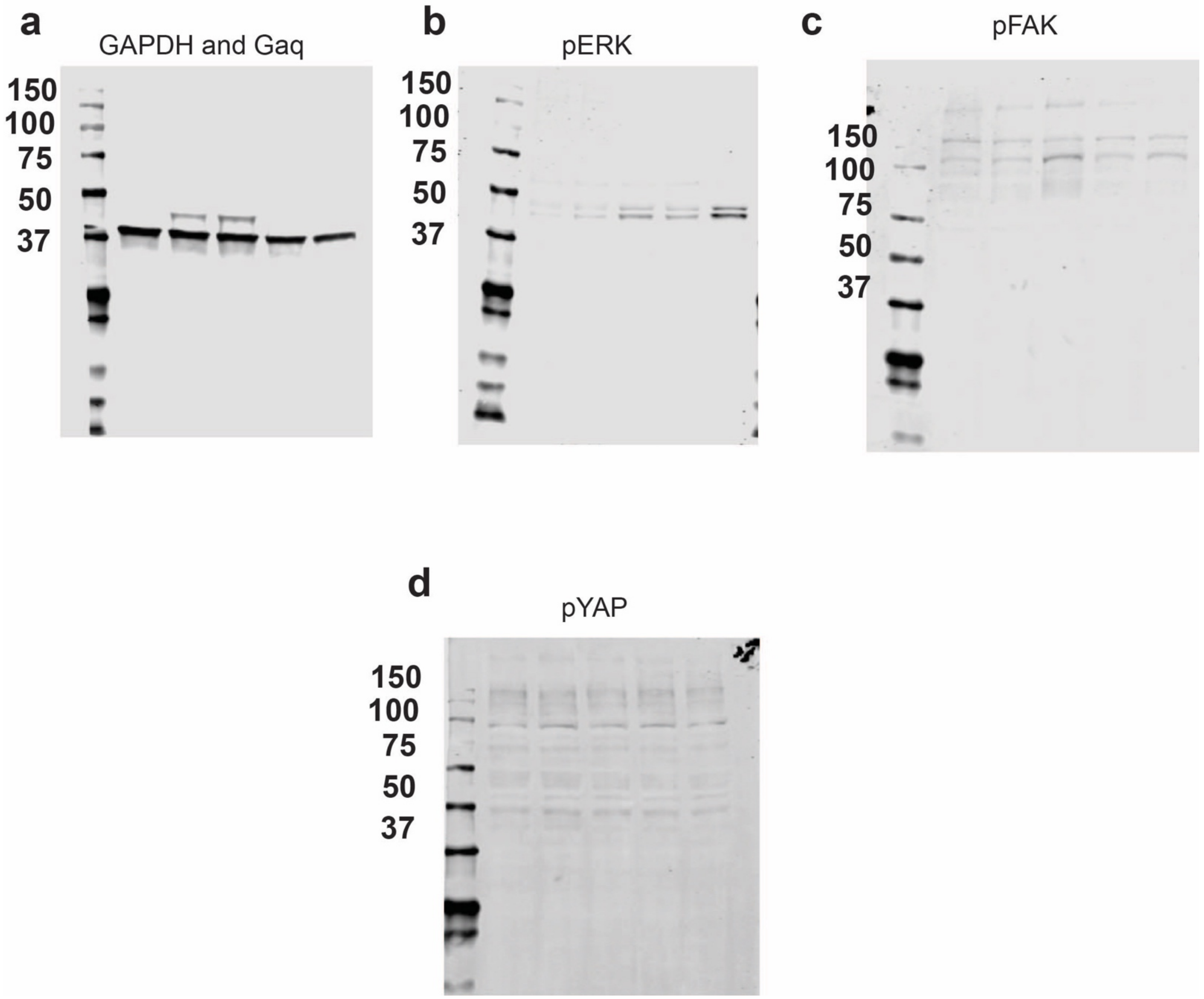

Source Data File. Uncropped blots that correspond with Figure 5c.

Supporting Information

Supplementary Model Rections and Equations Mechanistic reactions for model of WT GPCR signaling via $G\alpha_{q/11}$. For a schematic see Figure 1 of the main text.

$$\mathrm{G}_\alpha\mathrm{GDP} + \mathrm{G}_{\beta\gamma} \underset{k_{dG1}}{\overset{k_{G1}}{\rightleftharpoons}} \mathrm{G}$$

$$\mathrm{G} \xrightarrow{k_{cat1}[RL],K_{m1}} \mathrm{G}_\alpha\mathrm{GTP} + \mathrm{G}_{\beta\gamma}$$

$$\mathrm{G}_\alpha\mathrm{GTP} \xrightarrow{k_{hyd}} \mathrm{G}_\alpha\mathrm{GDP}$$

$$\mathrm{G}_\alpha\mathrm{GTP}\cdot\mathrm{TRIO} \xrightarrow{k_{hyd}} \mathrm{G}_\alpha\mathrm{GDP} + \mathrm{TRIO}$$

$$\mathrm{G}_\alpha\mathrm{GTP}\cdot\mathrm{PLC}\beta \xrightarrow{k_{hyd}} \mathrm{G}_\alpha\mathrm{GDP} + \mathrm{PLC}\beta$$

$$\mathrm{G}_\alpha\mathrm{GTP} + \mathrm{PLC}\beta \underset{k_{dt1}}{\overset{k_{at1}}{\rightleftharpoons}} \mathrm{G}_\alpha\mathrm{GTP}\cdot\mathrm{PLC}\beta$$

$$\mathrm{G}_\alpha\mathrm{GTP} + \mathrm{TRIO} \underset{k_{dt2}}{\overset{k_{at2}}{\rightleftharpoons}} \mathrm{G}_\alpha\mathrm{GTP}\cdot\mathrm{TRIO}$$

$$\mathrm{G}_\alpha\mathrm{NF} + \mathrm{GTP} \underset{k_{dGTP}}{\overset{k_{aGTP}}{\rightleftharpoons}} \mathrm{G}_\alpha\mathrm{GTP}$$

$$\mathrm{G}_\alpha\mathrm{NF} + \mathrm{GDP} \underset{k_{dGDP}}{\overset{k_{aGDP}}{\rightleftharpoons}} \mathrm{G}_\alpha\mathrm{GDP}$$

$$\mathrm{G}_\alpha\mathrm{GTP} + \mathrm{RGS} \underset{k_{dt3}}{\overset{k_{at3}}{\rightleftharpoons}} \mathrm{G}_\alpha\mathrm{GTP}\cdot\mathrm{RGS}$$

$$\mathrm{G}_\alpha\mathrm{GTP}\cdot\mathrm{RGS} \xrightarrow{k_{cat2}} \mathrm{G}_\alpha\mathrm{GDP} + \mathrm{RGS}$$

$$\mathrm{G}_\alpha\mathrm{GTP}\cdot\mathrm{RGS} \xrightarrow{k_{hyd}} \mathrm{G}_\alpha\mathrm{GDP} + \mathrm{RGS}$$

$$\mathrm{G}_\alpha\mathrm{GTP}\cdot\mathrm{PLC}\beta \xrightarrow{k_{hyd2}} \mathrm{G}_\alpha\mathrm{GDP} + \mathrm{PLC}\beta$$

$$
\begin{aligned}
R0 &= k_{dG1}[G] - k_{G1}[G_\alpha GDP][G_{\beta\gamma}] \\
R1 &= \frac{k_{cat1}[RL][G]}{K_{m1}(1 + [G^{mut}/K_{m1}]) + G} \\
R2 &= k_{hyd}[G_\alpha GTP] \\
R3 &= k_{hyd}[G_\alpha GTP \cdot TRIO] \\
R4 &= k_{hyd}[G_\alpha GTP \cdot PLC\beta] \\
R5 &= k_{dt1}[G_\alpha GTP \cdot PLC\beta] - k_{at1}[G_\alpha GTP][PLC\beta] \\
R6 &= k_{dt2}[G_\alpha GTP \cdot TRIO] - k_{at2}[G_\alpha GTP][TRIO] \\
R7 &= k_{dGTP}[G_\alpha GTP] - k_{aGTP}[GTP][G_\alpha NF] \\
R8 &= k_{dGDP}[G_\alpha GDP] - k_{aGDP}[GDP][G_\alpha NF] \\
R9 &= k_{dt3}[G_\alpha GTP \cdot RGS] - k_{at3}[G_\alpha GTP][RGS] \\
R10 &= k_{cat2}[G_\alpha GTP \cdot RGS] \\
R11 &= k_{hyd}[G_\alpha GTP \cdot RGS] \\
R12 &= k_{hyd2}[G_\alpha GTP \cdot PLC\beta]
\end{aligned}
$$

Corresponding ordinary differential equations (ODEs), in addition to the conservation relation: $[G_{\beta\gamma}] = G_0 - [G] - [G^{mut}]$

$$
\begin{aligned}
\frac{d[G]}{dt} &= -R0 - R1 \\
\frac{d[G_\alpha GTP]}{dt} &= R1 - R2 + R5 + R6 - R7 + R9 \\
\frac{d[G_\alpha GDP]}{dt} &= R0 + R2 + R3 + R4 - R8 + R10 + R11 + R12 \\
\frac{d[G_\alpha NF]}{dt} &= R7 + R8 \\
\frac{d[TRIO]}{dt} &= R3 + R6 \\
\frac{d[G_\alpha GTP \cdot TRIO]}{dt} &= -R3 - R6 \\
\frac{d[PLC\beta]}{dt} &= R4 + R5 + R12 \\
\frac{d[G_\alpha GTP \cdot PLC\beta]}{dt} &= -R4 - R5 - R12 \\
\frac{d[RGS]}{dt} &= R9 + R10 + R11 \\
\frac{d[G_\alpha GTP \cdot RGS]}{dt} &= -R9 - R10 - R11
\end{aligned}
$$

Initial Model Parameter Values

WT parameter values and references Table of the initial approximation of parameter values and references used in the model for the WT $G\alpha_{q/11}$ subunit reactions. These parameters were used in Figures 2-3 of the main text.

Parameter	Value	Description	Source
RL	$0.3\ \#/\mu m^2$	Amount activated receptor	[1–3]
GTP	$1.8 \cdot 10^{-4}\ M$	Amount GTP	[4]
GDP	$1.8 \cdot 10^{-5}\ M$	Amount GDP	[4]
RGS_0	$40\ \#/\mu m^2$	Initial concentration of RGS	[1,2]
G_0	$100\ \#/\mu m^2$	Initial concentration of G protein	[1,2]
$TRIO_0$	$10\ \#/\mu m^2$	Initial concentration of TRIO	[1,2]
$PLC\beta_0$	$100\ \#/\mu m^2$	Initial concentration of $PLC\beta$	[1,2]
k_{cat1}	$10.0\ s^{-1}$	GEF stimulation by activated receptor	[5]
K_{m1}	$250\ \#/\mu m^2$	GEF stimulation by activated receptor	[5]
k_{cat2}	$25.0\ s^{-1}$	GAP stimulation by RGS	[6]
k_{hyd}	$0.013\ s^{-1}$	GTPase activity of G_α	[7]
k_{hyd2}	$10.0\ s^{-1}$	GAP stimulation by $PLC\beta$	[6]
k_{aGTP}	$1.1 \cdot 10^5\ M^{-1}s^{-1}$	$G_\alpha NF$ bind GTP	[8]
k_{dGTP}	$1.3 \cdot 10^{-5}\ s^{-1}$	$G_\alpha GTP$ release of GTP	[8]
k_{aGDP}	$1.1 \cdot 10^5\ M^{-1}s^{-1}$	$G_\alpha NF$ bind GDP	[8]
k_{dGDP}	$3.5 \cdot 10^{-4}\ s^{-1}$	$G_\alpha GDP$ release of GDP	[8]
k_{G1}	$0.0825\ (\#/\mu m^2)^{-1}s^{-1}$	Recombination of $G_\alpha GDP$ and $G_{\beta\gamma}$	[5,9]
k_{dG1}	$0.0027\ s^{-1}$	Basal disassociation of G protein	[5,10]
k_{at1}	$0.498\ (\#/\mu m^2)^{-1}s^{-1}$	$G_\alpha GTP$ binding $PLC\beta$	[11]
k_{dt1}	$0.3\ s^{-1}$	Dissociation of $PLC\beta$ and G_α	[11]
k_{at2}	$0.498\ (\#/\mu m^2)^{-1}s^{-1}$	$G_\alpha GTP$ binding TRIO	[11,12]
k_{dt2}	$0.3\ s^{-1}$	Dissociation of TRIO and G_α	[11,13]
k_{at3}	$0.083\ (\#/\mu m^2)^{-1}s^{-1}$	$G_\alpha GTP$ binding RGS	[6]
k_{dt3}	$0.3\ s^{-1}$	Dissociation of RGS and G_α	[11,13]

Mutant species Mutant $G\alpha_q$ subunits are modeled explicitly with the appropriately adjusted reaction rate constants in addition to the reactions given above for the WT case. For example, for the Q209L GNAQ mutant we have the following to reflect the decreased rates of basal and GAP stimulated hydrolysis (see the tables below for the values of the mutant rate constants):

$$\begin{aligned}
R2^{mut} &= k_{hyd}^{mut}[G_\alpha^{mut}GTP] \\
R9^{mut} &= k_{dt3}[G_\alpha^{mut}GTP \cdot RGS] - k_{at3}^{mut}[G_\alpha^{mut}GTP][RGS] \\
R10^{mut} &= k_{cat2}^{mut}[G_\alpha^{mut}GTP \cdot RGS] \\
R11^{mut} &= k_{hyd}^{mut}[G_\alpha^{mut}GTP \cdot RGS] \\
R12^{mut} &= k_{hyd2}^{mut}[G_\alpha^{mut}GTP \cdot PLC\beta] \\
\frac{d[G_\alpha^{mut}GTP]}{dt} &= R1^{mut} - R2^{mut} + R5^{mut} + R6^{mut} - R7^{mut} + R9^{mut}
\end{aligned}$$

Q209L GNAQ parameter values and references The parameters that differ from WT. These parameters were used in Figures 2-3 of the main text.

Parameter	Value	Description	Source
k_{hyd}^{mut}	$k_{hyd}/140\ s^{-1}$	GTPase activity of G_α	[14–16]
k_{cat2}^{mut}	$k_{hyd}^{mut}\ s^{-1}$	GAP stimulation by RGS	[17, 18]
k_{hyd2}^{mut}	$k_{hyd}^{mut}\ s^{-1}$	GAP stimulation by $PLC\beta$	[17, 18]
k_{at1}^{mut}	$k_{at1} * 0.95\ (\#/\mu m^2)^{-1}s^{-1}$	$G_\alpha GTP$ binding $PLC\beta$	[16]
k_{at2}^{mut}	$k_{at2} * 1.1\ (\#/\mu m^2)^{-1}s^{-1}$	$G_\alpha GTP$ binding TRIO	[16]
k_{at3}^{mut}	$k_{at3} * 1.33\ (\#/\mu m^2)^{-1}s^{-1}$	$G_\alpha GTP$ binding RGS	[16]

Q209P GNAQ parameter values and references The parameters that differ from WT. These parameters were used in Figure 2 of the main text.

Parameter	Value	Description	Source
k_{hyd}^{mut}	$k_{hyd}/140\ s^{-1}$	GTPase activity of G_α	[14–16]
k_{cat2}^{mut}	$k_{hyd}^{mut}\ s^{-1}$	GAP stimulation by RGS	[17, 18]
k_{hyd2}^{mut}	$k_{hyd}^{mut}\ s^{-1}$	GAP stimulation by $PLC\beta$	[17, 18]
k_{at1}^{mut}	$k_{at1} * 0.66\ (\#/\mu m^2)^{-1}s^{-1}$	$G_\alpha GTP$ binding $PLC\beta$	[16]
k_{at2}^{mut}	$k_{at2} * 0.66\ (\#/\mu m^2)^{-1}s^{-1}$	$G_\alpha GTP$ binding TRIO	[16]
k_{at3}^{mut}	$k_{at3} * 0.66\ (\#/\mu m^2)^{-1}s^{-1}$	$G_\alpha GTP$ binding RGS	[16]

R183C GNAQ parameter values and references The parameters that differ from WT. These parameters were used in Figures 2-3 of the main text.

Parameter	Value	Description	Source
k_{hyd}^{mut}	$k_{hyd}/140\ s^{-1}$	GTPase activity of G_α	[14, 17]
k_{cat2}^{mut}	$k_{hyd}^{mut} * 110\ s^{-1}$	GAP stimulation by RGS	[14, 17]
k_{hyd2}^{mut}	$k_{hyd}^{mut} * 7\ s^{-1}$	GAP stimulation by $PLC\beta$	[14, 17]

L129Q CYSLTR2 parameter values and references The parameters that differ from WT. These parameters were used in Figure 2 of the main text.

Parameter	Value	Description	Source
RL^{mut}	$(RL/2) + (RL/2) * 13.6\ \#/\mu m^2$	Amount activated receptor	[3, 19]

Agonist stimulation parameter values and references The parameters that differ from basal. These parameters were used in Figure 2 of the main text for the agonist-stimulated case.

Parameter	Value	Description	Source
RL^{stim}	$RL * 6\ \#/\mu m^2$	Amount agonist-stimulated receptor	[3]

Model of FR drug mechanism Reactions to model the mechanism of the drug FR based on the current consensus of a GDI-like mechanism:

$$R11 = k_{dfr}[G_\alpha GDP \cdot FR] - k_{afr}[G_\alpha GDP][FR]$$
$$R12 = k_{dG1}[G \cdot FR] - k_{G1}[G_\alpha GDP \cdot FR][G_{\beta\gamma}]$$
$$R13 = k_{dfr}[G \cdot FR] - k_{afr}[G][FR]$$

FR model parameters The parameters for drug binding, assumed to be stronger than $G_{\beta\gamma}$ binding. These parameters were used in Figure 3 of the main text and Supplementary Figure 3.

Parameter	Value	Description	Source
k_{afr}	$k_{G1} * 10\ (\#/\mu m^2)^{-1} s^{-1}$	G_α binding FR	Assumption
k_{dfr}	$k_{dG1}\ s^{-1}$	Dissociation of G_α and FR	Assumption

Parameters varied for the parameter sweep and global sensitivity analysis Each parameter of the model varied for the sensitivity analysis. The number in the identifier is used to reference the parameter in Figure 4C of the main text and Supplementary Figure 2. The range of the parameters used in the Latin Hypercube Sampling for the parameter sweep and the Sobol sensitivity analysis, are indicated. The Kolmogorov-Smirnov (KS) statistic and p-value (via the Kolmogorov-Smirnov test) comparing the sampled input parameter distribution to the parameter distribution that matched the experiment.

Identifier	Parameter	Description	Range Varied Over	KS Statistic	KS p-value
1	G	Total amount of heterotrimeric G protein	[100,200] $\#/\mu m^2$	0.117	2.55e-6
2	RL	Basal amount of active receptor	[0.1,0.5] $\#/\mu m^2$	0.096	2.28e-4
3	$TRIO$	Total amount of $TRIO$	[10,100] $\#/\mu m^2$	0.128	1.80e-7
4	$PLC\beta$	Total amount of $PLC\beta$	[75,125] $\#/\mu m^2$	0.066	2.85e-2
5	RGS	Total amount of RGS	[40,100] $\#/\mu m^2$	0.060	5.97e-2
6	k_{at1}	$G_{\alpha q}GTP$ binding $PLC\beta$	[0.0498,4.98] $(\#/\mu m^2)^{-1}s^{-1}$	0.077	5.80e-3
7	k_{at2}	$G_{\alpha q}GTP$ binding $TRIO$	[0.0498,4.98] $(\#/\mu m^2)^{-1}s^{-1}$	0.117	2.67e-6
8	k_{at3}	$G_{\alpha q}GTP$ binding RGS	[0.0083,0.83] $(\#/\mu m^2)^{-1}s^{-1}$	0.127	2.58e-7
9	k_{hyd2}/k_{hyd}	Fold $PLC\beta$ hydrolysis	[1,100]	0.335	2.10e-48
10	$k_{hyd}^{mut}/k_{hyd}^{WT}$	Fold basal hydrolysis Q209L	[0.007,0.33]	0.133	5.08e-8
11	K_{m1}	GEF stimulation by active receptor	[50,500] $\#/\mu m^2$	0.106	2.88e-5
12	$k_{at1}^{mut}/k_{at1}^{WT}$	Bias of Q209L binding $PLC\beta$	[0.25,4]	0.672	7.60e-194
13	$k_{at2}^{mut}/k_{at2}^{WT}$	Bias of Q209L binding $TRIO$	[0.25,4]	0.425	9.82e-78

Updated Model Parameter Values

WT parameter values (updated) and references Table of the updated parameter values and references used in the model for the WT and Q209L $G\alpha_{q/11}$ subunit reactions. These parameters were used in Supplementary Figure 3 as an updated parameter set to achieve a ratio of mutant GNAQ binding of $k_{at2}^{mut}/k_{at1}^{mut} = 4$ and a fold $PLC\beta$ hydrolysis of $k_{hyd2}/k_{hyd} = 15$ based on parameter sweeps in Figure 6 of main text to align with the results of the experiment in Figure 5 of the main text. The parameters that are updated from the initial approximation above are in bold font while non-bolded parameters remain the same as the initial estimate. It should be noted that parameter values specific to the GNAQ Q209P and R183C mutants are left unchanged as we do not have any new experimental observations to base any potential updates on as we do for Q209L (how these mutants compare in downstream activation is a potential future direction for this modeling work). Similarly, in this updated parameterization, the parameters specific to CYSLTR2 L129Q are unchanged but the change in the fold $PLC\beta$ hydrolysis is critical for activation of WT $G_{\alpha q}$ downstream.

Parameter	Value	Description	Source
RL	$0.3\ \#/\mu m^2$	Amount activated receptor	[1–3]
GTP	$1.8 \cdot 10^{-4}\ M$	Amount GTP	[4]
GDP	$1.8 \cdot 10^{-5}\ M$	Amount GDP	[4]
RGS_0	$40\ \#/\mu m^2$	Initial concentration of RGS	[1, 2]
G_0	$100\ \#/\mu m^2$	Initial concentration of G protein	[1, 2]
$TRIO_0$	$10\ \#/\mu m^2$	Initial concentration of TRIO	[1, 2]
$PLC\beta_0$	$100\ \#/\mu m^2$	Initial concentration of $PLC\beta$	[1, 2]
k_{cat1}	$10.0\ s^{-1}$	GEF stimulation by activated receptor	[5]
K_{m1}	$250\ \#/\mu m^2$	GEF stimulation by activated receptor	[5]
k_{cat2}	$25.0\ s^{-1}$	GAP stimulation by RGS	[6]
k_{hyd}	$0.013\ s^{-1}$	GTPase activity of G_α	[7]
$\mathbf{k_{hyd2}}$	$\mathbf{k_{hyd} * 15\ s^{-1}}$	**GAP stimulation by PLC**β	**Figure 6 Main Text**
k_{aGTP}	$1.1 \cdot 10^5\ M^{-1}s^{-1}$	$G_\alpha NF$ bind GTP	[8]
k_{dGTP}	$1.3 \cdot 10^{-5}\ s^{-1}$	$G_\alpha GTP$ release of GTP	[8]
k_{aGDP}	$1.1 \cdot 10^5\ M^{-1}s^{-1}$	$G_\alpha NF$ bind GDP	[8]
k_{dGDP}	$3.5 \cdot 10^{-4}\ s^{-1}$	$G_\alpha GDP$ release of GDP	[8]
k_{G1}	$0.0825\ (\#/\mu m^2)^{-1}s^{-1}$	Recombination of $G_\alpha GDP$ and $G_{\beta\gamma}$	[5, 9]
k_{dG1}	$0.0027\ s^{-1}$	Basal disassociation of G protein	[5, 10]
k_{at1}	$0.498\ (\#/\mu m^2)^{-1}s^{-1}$	$G_\alpha GTP$ binding $PLC\beta$	[11]
k_{dt1}	$0.3\ s^{-1}$	Dissociation of $PLC\beta$ and G_α	[11]
k_{at2}	$0.498\ (\#/\mu m^2)^{-1}s^{-1}$	$G_\alpha GTP$ binding TRIO	[11, 12]
k_{dt2}	$0.3\ s^{-1}$	Dissociation of TRIO and G_α	[11, 13]
k_{at3}	$0.083\ (\#/\mu m^2)^{-1}s^{-1}$	$G_\alpha GTP$ binding RGS	[6]
k_{dt3}	$0.3\ s^{-1}$	Dissociation of RGS and G_α	[11, 13]

Q209L GNAQ parameter values (updated) and references The parameters that differ from WT. These parameters were used in Supplementary Figure 3. The parameters that are updated from the initial approximation above are in bold font while non-bolded parameters remain the same as the initial estimate.

Parameter	Value	Description	Source
k_{hyd}^{mut}	$k_{hyd}/140\ s^{-1}$	GTPase activity of G_α	[14–16]
k_{cat2}^{mut}	$k_{hyd}^{mut}\ s^{-1}$	GAP stimulation by RGS	[17, 18]
k_{hyd2}^{mut}	$k_{hyd}^{mut}\ s^{-1}$	GAP stimulation by $PLC\beta$	[17, 18]
$\mathbf{k_{at1}^{mut}}$	$\mathbf{k_{at1} * 0.5\ (\#/\mu m^2)^{-1}s^{-1}}$	**G_αGTP binding PLCβ**	**Figure 6 Main Text**
$\mathbf{k_{at2}^{mut}}$	$\mathbf{k_{at2} * 2\ (\#/\mu m^2)^{-1}s^{-1}}$	**G_αGTP binding TRIO**	**Figure 6 Main Text**
k_{at3}^{mut}	$k_{at3} * 1.33\ (\#/\mu m^2)^{-1}s^{-1}$	$G_\alpha GTP$ binding RGS	[16]

Q209P GNAQ parameter values (updated) and references The parameters that differ from WT. These parameters were used in Supplementary Figure 3. The parameters that are updated from the initial approximation above are in bold font while non-bolded parameters remain the same as the initial estimate.

Parameter	Value	Description	Source
k_{hyd}^{mut}	$k_{hyd}/140\ s^{-1}$	GTPase activity of G_α	[14–16]
k_{cat2}^{mut}	$k_{hyd}^{mut}\ s^{-1}$	GAP stimulation by RGS	[17, 18]
k_{hyd2}^{mut}	$k_{hyd}^{mut}\ s^{-1}$	GAP stimulation by $PLC\beta$	[17, 18]
k_{at1}^{mut}	$k_{at1} * 0.66\ (\#/\mu m^2)^{-1}s^{-1}$	$G_\alpha GTP$ binding $PLC\beta$	[16]
k_{at2}^{mut}	$k_{at2} * 0.66\ (\#/\mu m^2)^{-1}s^{-1}$	$G_\alpha GTP$ binding TRIO	[16]
k_{at3}^{mut}	$k_{at3} * 0.66\ (\#/\mu m^2)^{-1}s^{-1}$	$G_\alpha GTP$ binding RGS	[16]

R183C GNAQ parameter values (updated) and references The parameters that differ from WT. These parameters were used in Supplementary Figure 3. The parameters that are updated from the initial approximation above are in bold font while non-bolded parameters remain the same as the initial estimate.

Parameter	Value	Description	Source
k_{hyd}^{mut}	$k_{hyd}/140\ s^{-1}$	GTPase activity of G_α	[14,17]
k_{cat2}^{mut}	$k_{hyd}^{mut} * 110\ s^{-1}$	GAP stimulation by RGS	[14,17]
k_{hyd2}^{mut}	$k_{hyd}^{mut} * 7\ s^{-1}$	GAP stimulation by $PLC\beta$	[14,17]

L129Q CYSLTR2 parameter values (updated) and references The parameters that differ from WT. These parameters were used in Supplementary Figure 3. The parameters that are updated from the initial approximation above are in bold font while non-bolded parameters remain the same as the initial estimate.

Parameter	Value	Description	Source
RL^{mut}	$(RL/2) + (RL/2) * 13.6\ \#/\mu m^2$	Amount activated receptor	[3,19]

Note on model limitations It is important to note, as with every model system, our mathematical model has several limitations. One major challenge is the lack of standardized, consistent biochemical data on the rate constants and concentrations for both the WT and mutant proteins considered in this study. While there are several solid measurements in the literature for certain reactions, they are often done under different experimental conditions and only specific reactions or mutants are considered in a given study. To address these issues, we have tried to focus on reactions that are relatively well characterized and use the model to make predictions that are robust to changes in parameter values. Also, our model cannot consider every reaction relevant to a given pathway, as is always the case. Mechanisms that are beyond the scope of our model, such as β-arrestin and G-protein-coupled receptor kinase (GRK) signaling, receptor internalization, signaling from internal compartments and downstream dynamics likely play additional roles. Our approach was to leverage the model as a unique perspective on the potential behaviors of the core components of oncogenic signaling in UM and try to discern implications of the model that are potentially relevant for UM patients. This type of systems biology approach has emerged as a powerful tool to elucidate the systems-level consequences of oncogenic mutations and response to targeted therapy. In previous work, a mechanistic mathematical model of oncogenic RAS signaling helped provide insight into critical differences between classes of activating RAS point mutations and identified a mechanism to explain a confusing clinical observation regarding the response to targeted therapy [20, 21].

Supplementary References

1. Kulak NA, Pichler G, Paron I, Nagaraj N, Mann M. Minimal, encapsulated proteomic-sample processing applied to copy-number estimation in eukaryotic cells. Nature Methods. 2014;11:319 EP –.

2. Hein M, Hubner N, Poser I, Cox J, Nagaraj N, Toyoda Y, et al. A Human Interactome in Three Quantitative Dimensions Organized by Stoichiometries and Abundances. Cell. 2015;163(3):712 – 723. doi:https://doi.org/10.1016/j.cell.2015.09.053.

3. Wingler LM, Elgeti M, Hilger D, Latorraca NR, Lerch MT, Staus DP, et al. Angiotensin Analogs with Divergent Bias Stabilize Distinct Receptor Conformations. Cell. 2019;176(3):468 – 478.e11. doi:https://doi.org/10.1016/j.cell.2018.12.005.

4. Traut TW. Physiological concentrations of purines and pyrimidines. Molecular and Cellular Biochemistry. 1994;140(1):1–22. doi:10.1007/BF00928361.

5. Katanaev VL, Chornomorets M. Kinetic diversity in G-protein-coupled receptor signalling. Biochemical Journal. 2007;401(2):485–495. doi:10.1042/BJ20060517.

6. Mukhopadhyay S, Ross EM. Rapid GTP binding and hydrolysis by Gq promoted by receptor and GTPase-activating proteins. Proceedings of the National Academy of Sciences. 1999;96(17):9539–9544. doi:10.1073/pnas.96.17.9539.

7. Berstein G, Blank JL, Smrcka AV, Higashijima T, Sternweis PC, Exton JH, et al. Reconstitution of agonist-stimulated phosphatidylinositol 4,5-bisphosphate hydrolysis using purified m1 muscarinic receptor, Gq/11, and phospholipase C-beta 1. Journal of Biological Chemistry. 1992;267(12):8081–8.

8. Chidiac P, Markin VS, Ross EM. Kinetic control of guanine nucleotide binding to soluble Gaq. Biochemical Pharmacology. 1999;58(1):39 – 48. doi:https://doi.org/10.1016/S0006-2952(99)00080-5.

9. Sarvazyan NA, Lim WK, Neubig RR. Fluorescence Analysis of Receptor−G Protein Interactions in Cell Membranes. Biochemistry. 2002;41(42):12858–12867. doi:10.1021/bi026212l.

10. Sarvazyan NA, Remmers AE, Neubig RR. Determinants of Gi1a and Bg Binding: MEASURING HIGH AFFINITY INTERACTIONS IN A LIPID ENVIRONMENT USING FLOW CYTOMETRY. Journal of Biological Chemistry. 1998;273(14):7934–7940. doi:10.1074/jbc.273.14.7934.

11. Navaratnarajah P, Gershenson A, Ross EM. The binding of activated Gaq to phospholipase C-B exhibits anomalous affinity. Journal of Biological Chemistry. 2017;292(40):16787–16801. doi:10.1074/jbc.M117.809673.

12. Rojas RJ, Yohe ME, Gershburg S, Kawano T, Kozasa T, Sondek J. Gaq Directly Activates p63RhoGEF and Trio via a Conserved Extension of the Dbl Homology-associated Pleckstrin Homology Domain. Journal of Biological Chemistry. 2007;282(40):29201–29210. doi:10.1074/jbc.M703458200.

13. Bodmann EL, Rinne A, Brandt D, Lutz S, Wieland T, Grosse R, et al. Dynamics of Gaq-protein-p63RhoGEF interaction and its regulation by RGS2. Biochemical Journal. 2014;458(1):131–140. doi:10.1042/BJ20130782.

14. Chidiac P, Ross EM. Phospholipase C-B1 Directly Accelerates GTP Hydrolysis by Gaq and Acceleration Is Inhibited by GBg Subunits. Journal of Biological Chemistry. 1999;274(28):19639–19643. doi:10.1074/jbc.274.28.19639.

15. Kleuss C, Raw AS, Lee E, Sprang SR, Gilman AG. Mechanism of GTP hydrolysis by G-protein alpha subunits. Proceedings of the National Academy of Sciences. 1994;91(21):9828–9831. doi:10.1073/pnas.91.21.9828.

16. Maziarz M, Leyme A, Marivin A, Luebbers A, Patel PP, Chen Z, et al. Atypical activation of Gaq by the oncogenic mutation Q209P. Journal of Biological Chemistry. 2018;doi:10.1074/jbc.RA118.005291.

17. Berman DM, Wilkie TM, Gilman AG. GAIP and RGS4 Are GTPase-Activating Proteins for the Gi Subfamily of G Protein a Subunits. Cell. 1996;86(3):445 – 452. doi:https://doi.org/10.1016/S0092-8674(00)80117-8.

18. Anger T, Zhang W, Mende U. Differential Contribution of GTPase Activation and Effector Antagonism to the Inhibitory Effect of RGS Proteins on Gaq-mediated Signaling In Vivo. Journal of Biological Chemistry. 2004;279(6):3906–3915. doi:10.1074/jbc.M309496200.

19. Moore AR, Ceraudo E, Sher JJ, Guan Y, Shoushtari AN, Chang MT, et al. Recurrent activating mutations of G-protein-coupled receptor CYSLTR2 in uveal melanoma. Nature Genetics. 2016;48:675 EP –.

20. Stites EC, Trampont PC, Ma Z, Ravichandran KS. Network Analysis of Oncogenic Ras Activation in Cancer. Science. 2007;318(5849):463–467. doi:10.1126/science.1144642.

21. McFall T, Diedrich JK, Mengistu M, Littlechild SL, Paskvan KV, Sisk-Hackworth L, et al. A systems mechanism for KRAS mutant allele–specific responses to targeted therapy. Science Signaling. 2019;12(600). doi:10.1126/scisignal.aaw8288.
